# High-performance single-cell gene regulatory network inference at scale: the Inferelator 3.0

**DOI:** 10.1093/bioinformatics/btac117

**Published:** 2022-02-21

**Authors:** Claudia Skok Gibbs, Christopher A Jackson, Giuseppe-Antonio Saldi, Andreas Tjärnberg, Aashna Shah, Aaron Watters, Nicholas De Veaux, Konstantine Tchourine, Ren Yi, Tymor Hamamsy, Dayanne M Castro, Nicholas Carriero, Bram L Gorissen, David Gresham, Emily R Miraldi, Richard Bonneau

**Affiliations:** Flatiron Institute, Center for Computational Biology, Simons Foundation, New York, NY 10010, USA; Center for Data Science, New York University, New York, NY 10003, USA; Center for Genomics and Systems Biology, New York University, New York, NY 10003, USA; Department of Biology, New York University, New York, NY 10003, USA; Center for Genomics and Systems Biology, New York University, New York, NY 10003, USA; Department of Biology, New York University, New York, NY 10003, USA; Center for Genomics and Systems Biology, New York University, New York, NY 10003, USA; Department of Biology, New York University, New York, NY 10003, USA; Flatiron Institute, Center for Computational Biology, Simons Foundation, New York, NY 10010, USA; Flatiron Institute, Center for Computational Biology, Simons Foundation, New York, NY 10010, USA; Flatiron Institute, Center for Computational Biology, Simons Foundation, New York, NY 10010, USA; Department of Systems Biology, Columbia University, New York, NY 10027, USA; Computer Science Department, Courant Institute of Mathematical Sciences, New York University, New York, NY 10012, USA; Center for Data Science, New York University, New York, NY 10003, USA; Center for Genomics and Systems Biology, New York University, New York, NY 10003, USA; Department of Biology, New York University, New York, NY 10003, USA; Flatiron Institute, Scientific Computing Core, Simons Foundation, New York, NY 10010, USA; Stanley Center for Psychiatric Research, Broad Institute of MIT and Harvard, Cambridge, MA 02142, USA; Center for Genomics and Systems Biology, New York University, New York, NY 10003, USA; Department of Biology, New York University, New York, NY 10003, USA; Divisions of Immunobiology and Biomedical Informatics, Cincinnati Children’s Hospital Medical Center, Cincinnati, OH 45229, USA; Department of Pediatrics, University of Cincinnati College of Medicine, Cincinnati, OH 45267, USA; Flatiron Institute, Center for Computational Biology, Simons Foundation, New York, NY 10010, USA; Center for Data Science, New York University, New York, NY 10003, USA; Center for Genomics and Systems Biology, New York University, New York, NY 10003, USA; Department of Biology, New York University, New York, NY 10003, USA; Computer Science Department, Courant Institute of Mathematical Sciences, New York University, New York, NY 10012, USA

## Abstract

**Motivation:**

Gene regulatory networks define regulatory relationships between transcription factors and target genes within a biological system, and reconstructing them is essential for understanding cellular growth and function. Methods for inferring and reconstructing networks from genomics data have evolved rapidly over the last decade in response to advances in sequencing technology and machine learning. The scale of data collection has increased dramatically; the largest genome-wide gene expression datasets have grown from thousands of measurements to millions of single cells, and new technologies are on the horizon to increase to tens of millions of cells and above.

**Results:**

In this work, we present the Inferelator 3.0, which has been significantly updated to integrate data from distinct cell types to learn context-specific regulatory networks and aggregate them into a shared regulatory network, while retaining the functionality of the previous versions. The Inferelator is able to integrate the largest single-cell datasets and learn cell-type-specific gene regulatory networks. Compared to other network inference methods, the Inferelator learns new and informative *Saccharomyces cerevisiae* networks from single-cell gene expression data, measured by recovery of a known gold standard. We demonstrate its scaling capabilities by learning networks for multiple distinct neuronal and glial cell types in the developing *Mus musculus* brain at E18 from a large (1.3 million) single-cell gene expression dataset with paired single-cell chromatin accessibility data.

**Availability and implementation:**

The inferelator software is available on GitHub (https://github.com/flatironinstitute/inferelator) under the MIT license and has been released as python packages with associated documentation (https://inferelator.readthedocs.io/).

**Supplementary information:**

Supplementary data are available at *Bioinformatics* online.

## 1 Background

**Fig. 1.**
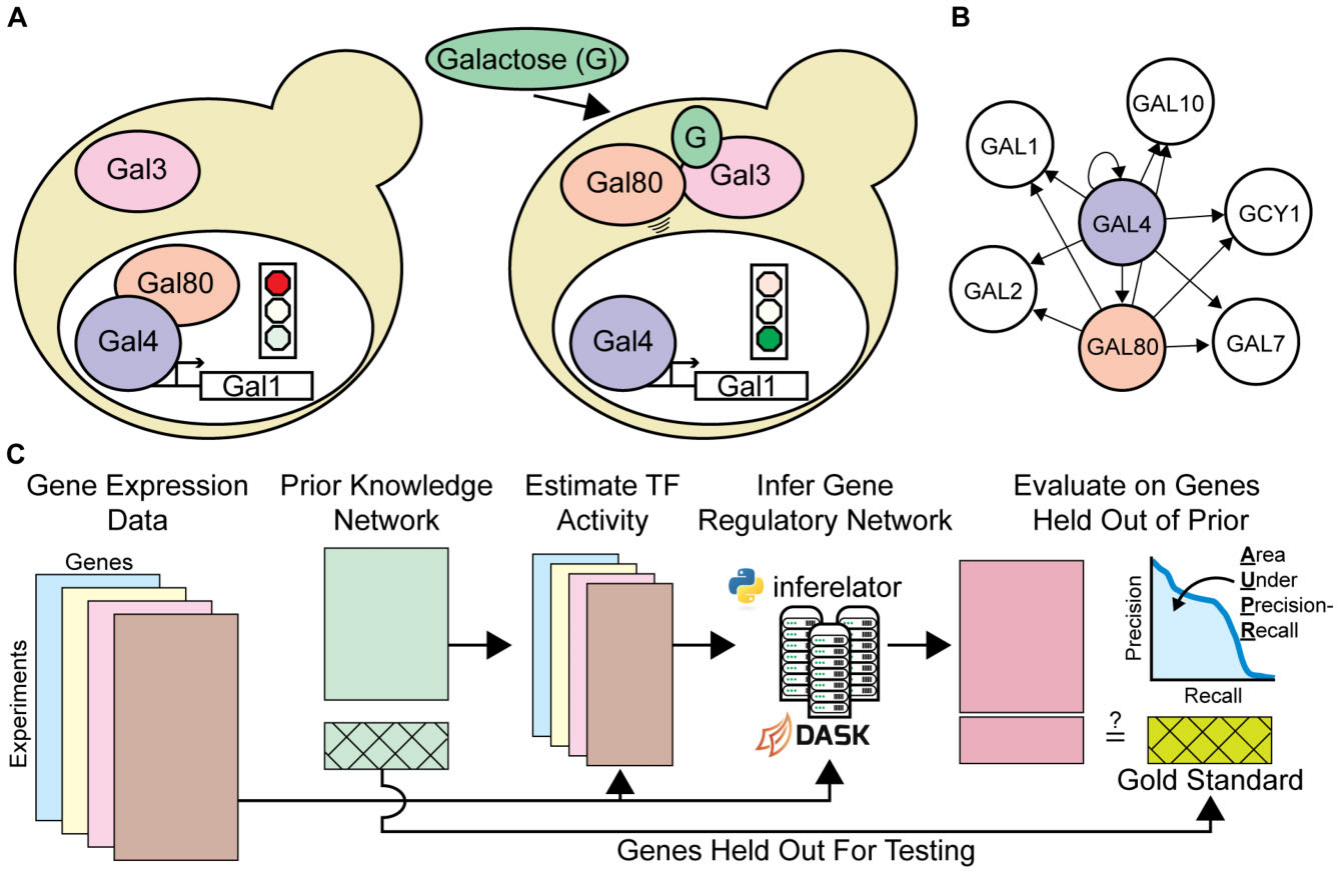
Learning GRNs with the Inferelator (**A**) The response to the sugar galactose in *S.cerevisiae* is mediated by the Gal4 and Gal80 TFs, a prototypical mechanism for altering cellular gene expression in response to stimuli. (**B**) Gal4 and Gal80 regulation represented as an unsigned directed graph connecting regulatory TFs to target genes. (**C**) Genome-wide GRNs are inferred from gene expression data and prior knowledge about network connections using the Inferelator, and the resulting networks are scored by comparison with a gold standard of known interactions. A subset of genes are held out of the prior knowledge and used for evaluating performance

Gene expression is tightly regulated at multiple levels in order to control cell growth, development and response to environmental conditions (Fig. 1A). Transcriptional regulation is principally controlled by transcription factors (TFs) that bind to DNA and effect chromatin remodeling (Zaret, 2020) or directly modulate the output of RNA polymerases (Kadonaga, 2004). About 3% of *Saccharomyces cerevisiae* genes are TFs (Hahn and Young, 2011), and more than 6% of human genes are believed to be TFs or cofactors (Lambert *et al.*, 2018). Connections between TFs and genes combine to form a transcriptional Gene Regulatory Network (GRN) that can be represented as a directed graph (Fig. 1B). Learning the true regulatory network that connects regulatory TFs to target genes is a key problem in biology (Chasman *et al.*, 2016; Thompson *et al.*, 2015). Determining the valid GRN is necessary to explain how mutations that cause gene dysregulation lead to complex disease states (Hu *et al.*, 2016), how variation at the genetic level leads to phenotypic variation (Mehta *et al.*, 2021; Peter and Davidson, 2011), and how to re-engineer organisms to efficiently produce industrial chemicals and enzymes (Huang *et al.*, 2017).

Learning genome-scale networks relies on genome-wide expression measurements, initially captured with microarray technology (DeRisi *et al.*, 1997), but today typically measured by RNA-sequencing (RNA-seq) (Nagalakshmi *et al.*, 2008). A major difficulty is that biological systems have large numbers of both regulators and targets, and many regulators are redundant or interdependent. Many plausible networks can explain observed expression data and the regulation of gene expression (Szederkényi *et al.*, 2011), which makes identifying the correct network challenging. Designing experiments to produce data that increases network identifiability is possible (Ud-Dean and Gunawan, 2016), but most data are collected for specific projects and repurposed for network inference as a consequence of the cost of data collection. Large-scale experiments in which a perturbation is made and dynamic data are collected over time is exceptionally useful for learning GRNs but systematic studies that collect this data are rare (Hackett *et al.*, 2020).

Measuring the expression of single cells using single-cell RNA-sequencing (scRNAseq) is an emerging and highly scalable technology. Microfluidic-based single-cell techniques (Macosko *et al.*, 2015; Zheng *et al.*, 2017; Zilionis *et al.*, 2017) allow for thousands of measurements in a single experiment. Split-pool barcoding techniques (Rosenberg *et al.*, 2018) are poised to increase single-cell throughput by an order of magnitude. These techniques have been successfully applied to generate multiplexed gene expression data from pools of barcoded cell lines with loss-of-function TF mutants (Dixit *et al.*, 2016; Jackson *et al.*, 2020), enhancer perturbations (Schraivogel *et al.*, 2020) and disease-causing oncogene variants (Ursu *et al.*, 2020). Individual cell measurements are sparser and noisier than measurements generated using traditional RNA-seq, although in aggregate the gene expression profiles of single-cell data match RNA-seq data well (Svensson, 2020), and techniques to denoise single-cell data have been developed (Arisdakessian *et al.*, 2019; Tjärnberg *et al.*, 2021).

The seurat (Stuart *et al.*, 2019) and scanpy (Wolf *et al.*, 2018) bioinformatics toolkits are established tools for single-cell data analysis, but pipelines for inferring GRNs from single-cell data are still nascent, although many are under development (Zappia and Theis, 2021). Recent work has begun to systematically benchmarking network inference tools, and the BEELINE (Pratapa *et al.*, 2020) and other (Chen and Mar, 2018; Nguyen *et al.*, 2021) benchmarks have identified promising methods. Testing on real-world data has proved difficult, as reliable gold standard networks for higher eukaryotes do not exist. scRNAseq data for microbes, which have some known ground truth networks (like *S.cerevisiae* and *Bacillus subtilis*) was not collected until recently. As a consequence, most computational method benchmarking has been done using simulated data. Finally, GRN inference is computationally challenging, and the most scalable currently-published GRN pipeline has learned GRNs from 50 000 cells of gene expression data (Van de Sande *et al.*, 2020).

Here we describe the Inferelator 3.0 pipeline for single-cell GRN inference, based on regularized regression (Bonneau *et al.*, 2006). This pipeline calculates TF activity (Ma and Brent, 2021) using a prior knowledge network and regresses scRNAseq expression data against that activity estimate to learn new regulatory edges. We compare it directly to two other network inference methods that also utilize prior network information and scRNAseq data, benchmarking using real-world *S.cerevisiae* scRNAseq data and comparing to a high-quality gold standard network. The first comparable method, SCENIC (Van de Sande *et al.*, 2020), is GRN inference pipeline that estimates the importance of TFs in explaining gene expression profiles and then constrains this correlative measure with prior network information to identify regulons. The second comparable method, CellOracle (Kamimoto *et al.*, 2020), has been recently proposed as a pipeline to integrate single-cell Assay for Transposase-Accessible Chromatin (ATAC) and expression data using a motif-based search for potential regulators, followed by bagging Bayesian ridge regression to enforce sparsity in the output GRN.

Older versions of the Inferelator (Madar *et al.*, 2009) have performed well inferring networks for *B.subtilis* (Arrieta-Ortiz *et al.*, 2015), human Th17 cells (Ciofani *et al.*, 2012; Miraldi *et al.*, 2019), mouse lymphocytes (Pokrovskii *et al.*, 2019), *S.cerevisiae* (Tchourine *et al.*, 2018) and *Oryza sativa* (Wilkins *et al.*, 2016). We have implemented the Inferelator 3.0 with new functionality in python to learn GRNs from scRNAseq data. Three different model selection methods have been implemented: a Bayesian best-subset regression (BBSR) method (Greenfield *et al.*, 2013), a Stability Approach to Regularization Selection for Least Absolute Shrinkage and Selection Operator (StARS-LASSO) (Miraldi *et al.*, 2019) regression method in which the regularization parameter is set by stability selection (Liu *et al.*, 2010) and a multi-task-learning regression method (Castro *et al.*, 2019). This new package provides scalability, allowing millions of cells to be analyzed together, as well as integrated support for multi-task GRN inference, while retaining the ability to utilize bulk gene expression data. We show that the Inferelator 3.0 is a state-of-the-art method by testing against SCENIC and CellOracle on model organisms with reliable ground truth networks, and show that the Inferelator 3.0 can generate a mouse neuronal GRN from a publicly available dataset containing 1.3 million cells.

## 2 Results

### 2.1 The Inferelator 3.0

In the 12 years since the last major release of the Inferelator (Madar *et al.*, 2009), the scale of data collection in biology has accelerated enormously. We have therefore rewritten the Inferelator as a python package to take advantage of the concurrent advances in data processing. For inference from small-scale gene expression datasets ($<10^{4}$ observations), the Inferelator 3.0 uses native python multiprocessing to run on individual computers. For inference from extremely large-scale gene expression datasets ( $>10^{4}$ observations) that are increasingly available from scRNAseq experiments, the Inferelator 3.0 takes advantage of the Dask analytic engine (Rocklin, 2015) for deployment to high-performance clusters (Fig. 1C), or for deployment as a kubernetes image to the Google cloud computing infrastructure.

### 2.2 Network inference using bulk RNA-Seq expression data

**Fig. 2.**
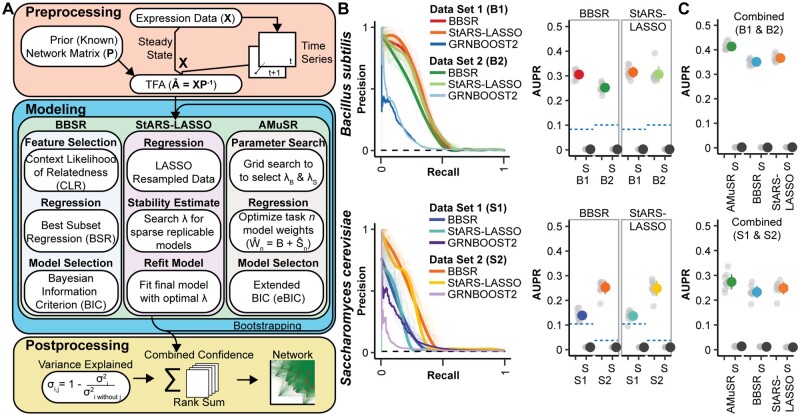
Network inference performance on multiple model organism datasets. (**A**) Schematic of Inferelator workflow and a brief summary of the differences between GRN model selection methods. (**B**) Results from 10 replicates of GRN inference for each modeling method on (i) *B.subtilis* GSE67023 (**B1**), GSE27219 (**B2**) and (ii) *S.cerevisiae* GSE142864 (S1), and Tchourine *et al.* (2018) (S2). Precision–recall curves are shown for replicates where 20% of genes are held out of the prior and used for evaluation, with a smoothed consensus curve. The black dashed line on the precision–recall curve is the expected random performance based on random sampling from the gold standard. AUPR is plotted for each cross-validation result in gray, with mean ± standard deviation in color. Experiments labeled with (S) are shuffled controls, where the labels on the prior adjacency matrix have been randomly shuffled. A total of 10 shuffled replicates are shown as gray dots, with mean ± standard deviation in black. The blue dashed line is the performance of the GRNBOOST2 network inference algorithm, which does not use prior network information, scored against the entire gold standard network. (**C**) Results from 10 replicates of GRN inference using two datasets as two network inference tasks on (i) *B.subtilis* and (ii) *S.cerevisiae*. AMuSR is a multi-task-learning method; BBSR and StARS-LASSO are run on each task separately and then combined into a unified GRN. AUPR is plotted as in (B)

We incorporated several network inference model selection methods into the Inferelator 3.0 (Fig. 2A) and evaluate their performance on the prokaryotic model *B.subtilis* and the eukaryotic model *S.cerevisiae*. Both *B.subtilis* (Arrieta-Ortiz *et al.*, 2015; Nicolas *et al.*, 2012) and *S.cerevisiae* (Hackett *et al.*, 2020; Tchourine *et al.*, 2018) have large bulk RNA-seq and microarray gene expression datasets, in addition to a relatively large number of experimentally determined TF–target gene interactions that can be used as a gold standard for assessing network inference. Using two independent datasets for each organism, we find that the model selection methods BBSR (Greenfield *et al.*, 2010) and StARS-LASSO (Miraldi *et al.*, 2019) perform equivalently (Fig. 2B). The Inferelator performs substantially better than a network inference method (GRNBOOST2) that does not use prior network information (Fig. 2B; dashed blue lines).

The two independent datasets show clear batch effects (Supplementary Fig. S1A), and combining them for network inference is difficult; conceptually, each dataset is in a separate space, and must be mapped into a shared space. We take a different approach to addressing the batch effects between datasets by treating them as separate learning tasks (Castro *et al.*, 2019) and then combining network information into a unified GRN. This results in a considerable improvement in network inference performance over either dataset individually (Fig. 2C). The best performance is obtained with Adaptive Multiple Sparse Regression (AMuSR) (Castro *et al.*, 2019), a multi-task learning method that shares information between tasks during regression. The GRN learned with AMuSR explains the variance in the expression data better than learning networks from each dataset individually with BBSR or StARS-LASSO and then combining them (Supplementary Fig. S1B), and retains a common network core across different tasks (Supplementary Fig. S1C).

### 2.3 Generating prior networks from chromatin data and TF motifs

The Inferelator 3.0 produces an inferred network from a combination of gene expression data and a prior knowledge GRN constructed from existing knowledge about known gene regulation. Curated databases of regulator–gene interactions culled from domain-specific literature are an excellent source for prior networks. While some model systems have excellent databases of known interactions, these resources are unavailable for most organisms or cell types. In these cases, using chromatin accessibility determined by a standard ATAC in combination with the known DNA-binding preferences for TFs to identify putative target genes is a viable alternative (Miraldi *et al.*, 2019).

**Fig. 3.**
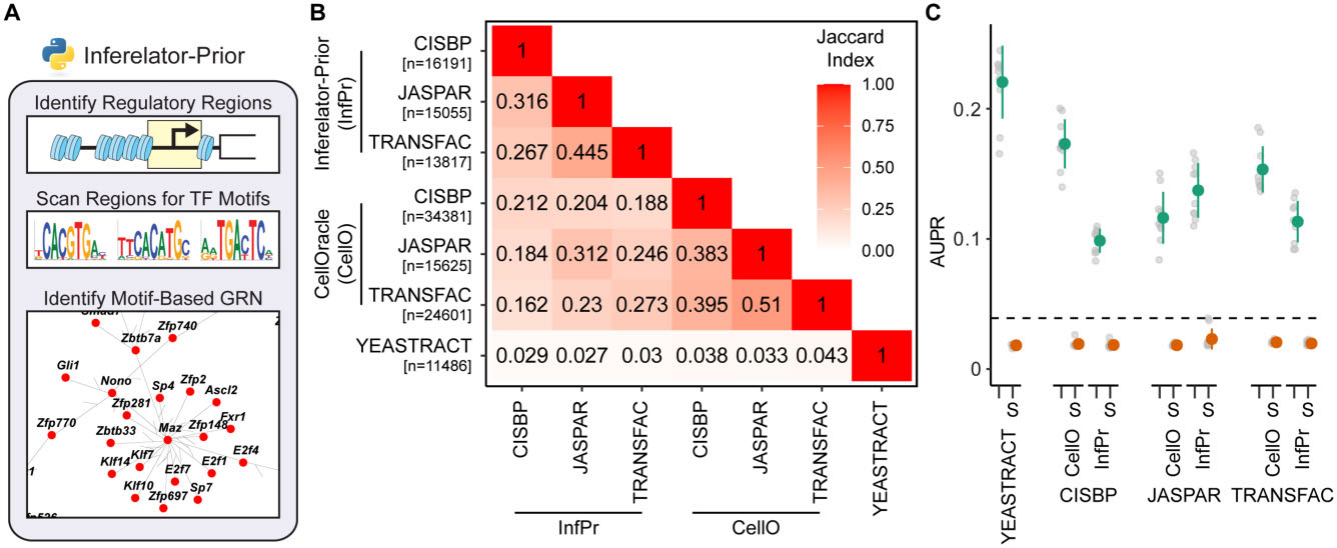
Construction and performance of network connectivity priors using TF motif scanning. (**A**) Schematic of inferelator-prior workflow, scanning identified regulatory regions (e.g. by ATAC) for TF motifs to construct adjacency matrices. (**B**) Jaccard similarity index between *S.cerevisiae* prior adjacency matrices generated by the inferelator-prior package, by the CellOracle package, and obtained from the YEASTRACT database. Prior matrices were generated using TF motifs from the CIS-BP, JASPAR and TRANSFAC databases with each pipeline (*n* is the number of edges in each prior adjacency matrix). (**C**) The performance of Inferelator network inference using each motif-derived prior. Performance is evaluated by AUPR, scoring against genes held out of the prior adjacency matrix, based on inference using 2577 genome-wide microarray experiments. Experiments labeled with (S) are shuffled controls, where the labels on the prior adjacency matrix have been randomly shuffled. The black dashed line is the performance of the GRNBOOST2 algorithm, which does not incorporate prior knowledge, scored against the entire gold standard network

To generate these prior networks, we have developed the inferelator-prior accessory package that uses TF motif position-weight matrices to score TF binding within gene regulatory regions and build sparse prior networks (Fig. 3A). These gene regulatory regions can be identified by ATAC, by existing knowledge from TF Chromatin Immunoprecipitation experiments, or from known databases [e.g. ENCODE (ENCODE Project Consortium *et al.*, 2020)]. Here, we compare the inferelator-prior tool to the CellOracle package (Kamimoto *et al.*, 2020) that also constructs motif-based networks that can be constrained to regulatory regions, in *S.cerevisiae* by using sequences 200 bp upstream and 50 bp downstream of each gene TSS as the gene regulatory region. The inferelator-prior and CellOracle methods produce networks that are similar when measured by Jaccard index but are dissimilar to the YEASTRACT literature-derived network (Fig. 3B). These motif-derived prior networks from both the inferelator-prior and CellOracle methods perform well as prior knowledge for GRN inference using the Inferelator 3.0 pipeline (Fig. 3C). The source of the motif library has a significant effect on network output, as can be seen with the well-characterized TF GAL4. GAL4 has a canonical $\mathrm{CGG}N_{11}CGG$ binding site; different motif libraries have different annotated binding sites (Supplementary Fig. S2A) and yield different motif-derived networks with the inferelator-prior pipeline (Supplementary Fig. S2B and C).

### 2.4 Network inference using single-cell expression data

**Fig. 4.**
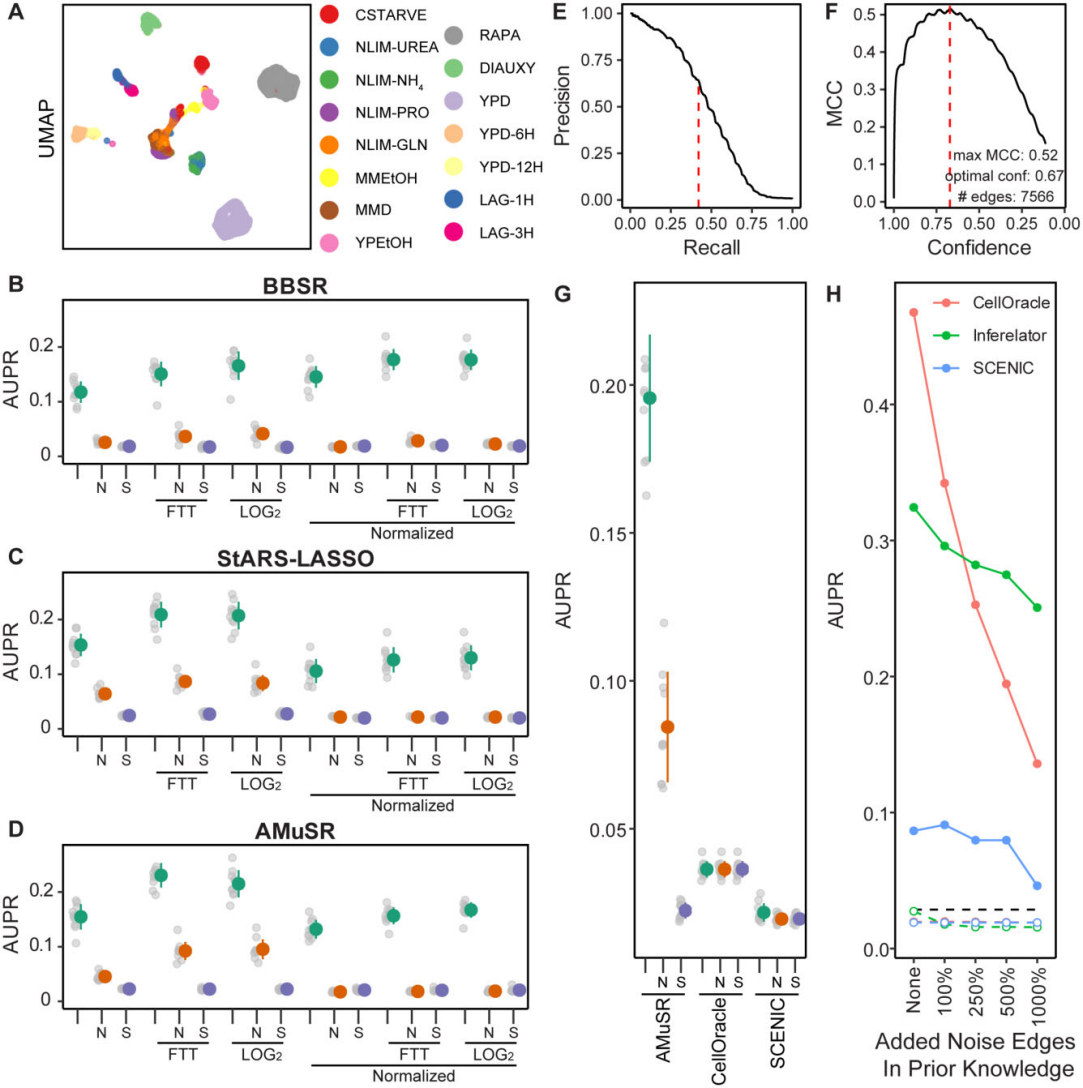
Network inference performance using *S.cerevisiae* single-cell data. (**A**) Uniform Manifold Approximation and Projection plot of yeast scRNAseq data, colored by the experimental grouping of individual cells (tasks). (**B**) The effect of preprocessing methods on network inference using BBSR model selection on 14 task-specific expression datasets, as measured by AUPR. Colored dots represent mean ± standard deviation of all replicates. Data are either untransformed (raw counts), transformed by Freeman–Tukey Transform (FTT), or transformed by $log_{2}(x\;+\;1)$ pseudocount. Non-normalized data are compared to data normalized so that all cells have identical count depth. Network inference performance is compared to two baseline controls; data, which have been replaced by Gaussian noise (N) and network inference using shuffled labels in the prior network (S). (**C**) Performance evaluated as in (B) on StARS-LASSO model selection. (**D**) Performance evaluated as in (B) on AMuSR model selection. (**E**) Precision–recall of a network constructed using FTT-transformed, non-normalized AMuSR model selection, as determined by the recovery of the prior network. Dashed red line is the retention threshold identified by MCC. (**F**) MCC of the same network as in (E). Dashed red line is the confidence score of the maximum MCC. (**G**) Performance evaluated as in (B) comparing the Inferelator (FTT-transformed, non-normalized AMuSR) against the SCENIC and CellOracle network inference pipelines. (**H**) Performance of the Inferelator (FTT-transformed, non-normalized AMuSR) compared to SCENIC and CellOracle without holding genes out of the prior knowledge network. Additional edges are added randomly to the prior knowledge network as a percentage of the true edges in the prior. Colored dashed lines represent controls for each method where the labels on the prior knowledge network are randomly shuffled. The black dashed line represents performance of the GRNBOOST2 algorithm, which identifies gene adjacencies as the first part of the SCENIC pipeline without using prior knowledge

Single-cell data are undersampled and noisy, but large numbers of observations are collected in parallel. As network inference is a population-level analysis, which must already be robust against noise, we reason that data preprocessing that improves per-cell analyses (like imputation) is unnecessary. We test this by quantitatively evaluating networks learned from *S.cerevisiae* scRNAseq data (Jackson *et al.*, 2020; Jariani *et al.*, 2020) with a previously-defined yeast gold standard (Tchourine *et al.*, 2018). This expression data is split into 15 separate tasks, based on labels that correspond to experimental conditions from the original works (Fig. 4A). A network is learned for each task separately using the YEASTRACT literature-derived prior network, from which a subset of genes are withheld, and aggregated into a final network for scoring on held-out genes from the gold standard. We test a combination of several preprocessing options with three network inference model selection methods (Fig. 4B–D).

We find that network inference is generally sensitive to the preprocessing options chosen, and that this effect outweighs the differences between different model selection methods (Fig. 4B–D). A standard Freeman–Tukey or log_2_ pseudocount transformation on raw count data yields the best performance, with notable decreases in recovery of the gold standard when count data are count depth normalized (such that each cell has the same total transcript counts). The performance of the randomly generated Noise control (*N*) is higher than the performance of the shuffled (*S*) control when counts per cell are not normalized, suggesting that total counts per cell provides additional information during inference.

Different model performance metrics, like area under the precision–recall (AUPR), Matthews Correlation Coefficient (MCC), and *F*1 score correlate very well and identify the same optimal hyperparameters (Supplementary Fig. S4). We apply AMuSR to data that has been Freeman–Tukey transformed to generate a final network without holding out genes for cross-validation (Fig. 4E). While we use AUPR as a metric for evaluating model performance, selecting a threshold for including edges in a GRN by precision or recall requires a target precision or recall to be chosen arbitrarily. Choosing the Inferelator confidence score threshold to include the edges in a final network that maximize MCC is a simple heuristic to select the size of a learned network that maximizes overlap with another network (e.g. a prior knowledge GRN or gold standard GRN) while minimizing links not in that network (Fig. 4F). Maximum *F*1 score gives a less conservative GRN as true negatives are not considered and will not diminish the score. Both metrics balance similarity to the test network with overall network size, and therefore represent straightforward heuristics that do not rely on arbitrary thresholds.

In order to determine how the Inferelator 3.0 compares to similar network inference tools, we apply both CellOracle and SCENIC to the same network inference problem, where a set of genes are held out of the prior knowledge GRN and used for scoring. We see that the Inferelator 3.0 can make predictions on genes for which no prior information is known, but CellOracle and SCENIC cannot (Fig. 4G). When provided with a complete prior knowledge GRN, testing on genes, which are not held out, CellOracle outperforms the Inferelator, although the Inferelator is more robust to noise in the prior knowledge GRN (Fig. 4H). This is a key advantage, as motif-generated prior knowledge GRNs are expected to be noisy.

### 2.5 Large-scale single-cell mouse neuron network inference

**Fig. 5.**
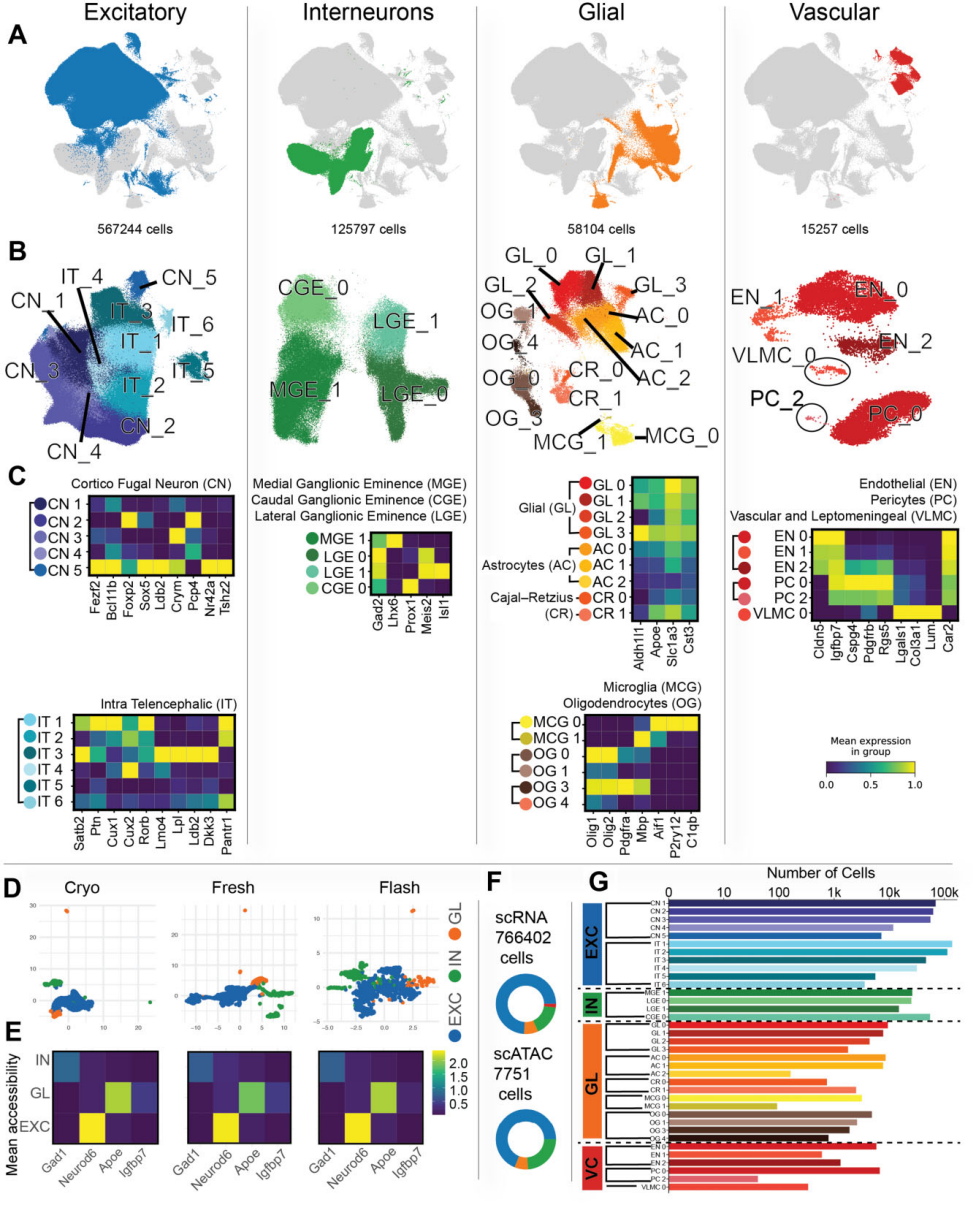
Processing large single-cell mouse brain data for network inference (**A**) Uniform Manifold Approximation and Projection plot of all mouse brain scRNAseq data with excitatory neurons, interneurons, glial cells and vascular cells colored. (**B**) Uniform Manifold Approximation and Projection plot of cells from each broad category colored by Louvain clusters and labeled by cell type. (**C**) Heatmap of normalized gene expression for marker genes that distinguish cluster cell types within broad categories. (**D**) Uniform Manifold Approximation and Projection plot of mouse brain scATAC data with excitatory neurons, interneurons and glial cells colored. (**E**) Heatmap of normalized mean gene accessibility for marker genes that distinguish broad categories of cells. (**F**) The number of scRNAseq and scATAC cells in each of the broad categories. (**G**) The number of scRNAseq cells in each cell-type-specific cluster

The Inferelator 3.0 is able to distribute work across multiple computational nodes, allowing networks to be rapidly learned from $>10^{5}$ cells (Supplementary Fig. S5A). We show this by applying the Inferelator to a large (1.3 million cells of scRNAseq data), publicly available dataset of mouse brain cells (10× genomics) that is accompanied by 15 000 single-cell ATAC (scATAC) measurements. We separate the expression and scATAC data into broad categories; excitatory neurons, interneurons, glial cells and vascular cells (Fig. 5A–E). After initial quality control, filtering and cell-type assignment, 766 402 scRNAseq and 7751 scATAC observations remain (Fig. 5F and Supplementary Fig. S5B–D).

scRNAseq data are further clustered within broad categories into clusters (Fig. 5B) that are assigned to specific cell types based on marker expression (Fig. 5C and Supplementary Fig. S6). scATAC data are aggregated into chromatin accessibility profiles for Excitatory neurons, Interneurons and Glial cells (Fig. 5D) based on accessibility profiles (Fig. 5E), which are then used with the TRANSFAC mouse motif position-weight matrices to construct prior knowledge GRNs with the inferelator-prior pipeline. Most scRNAseq cell-type clusters have thousands of cells;; however, rare cell-type clusters are smaller (Fig. 5G)

**Fig. 6.**
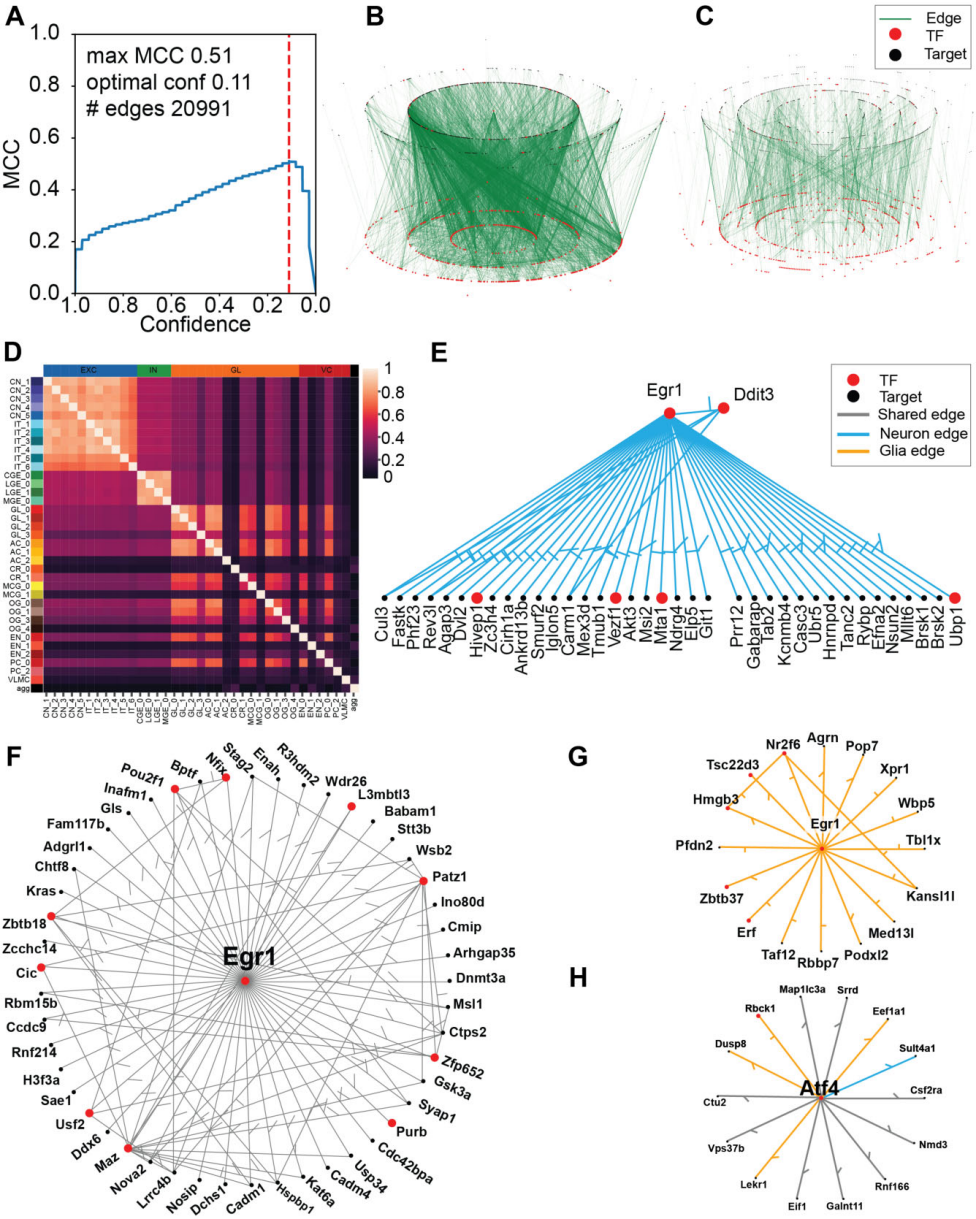
Learned GRN for the mouse brain (**A**) MCC for the aggregate network based on Inferelator prediction confidence. The dashed line shows the confidence score which maximizes MCC. Network edges at and above this line are retained in the final network. (**B**) Aggregate GRN learned. (**C**) Network edges, which are present in every individual task. (**D**) Jaccard similarity index between each task network. (**E**) Network targets of the *EGR1* TF in neurons. (**F**) Network targets of the *EGR1* TF in both neurons and glial cells. (**G**) Network targets of the *EGR1* TF in glial cells. (**H**) Network of the *ATF4* TF where blue edges are neuron specific, orange edges are glial specific and black edges are present in both categories

After processing scRNAseq into 36 cell-type clusters and scATAC data into three broad (Excitatory neurons, Interneurons and Glial) prior GRNs, we used the Inferelator 3.0 to learn an aggregate mouse brain GRN. Each of the 36 clusters was assigned the most appropriate of the three prior GRNs and learned as a separate task using the AMuSR model selection framework. The resulting aggregate network contains 20 991 TF–gene regulatory edges, selected from the highest-confidence predictions to maximize MCC (Fig. 6A and B). A common regulatory core of 1909 network edges is present in every task-specific network (Fig. 6C). Task-specific networks from similar cell types tend to be highly similar, as measured by Jaccard index (Fig. 6D). We learn very similar GRNs from each excitatory neuron task, and very similar GRNs from each interneuron task, although each of these broad categories yields different regulatory networks. There are also notable examples where glial and vascular tasks produce GRNs that are distinctively different from other glial and vascular GRNs.

Finally, we can examine specific TFs and compare networks between cell-type categories (Supplementary Fig. S7). The TFs Egr1 and Atf4 are expressed in all cell types and Egr1 is known to have an active role at embryonic day 18 (Sun *et al.*, 2019). In our learned network, Egr1 targets 103 genes, of which 20 are other TFs (Fig. 6E–G). Half of these targets (49) are common to both neurons and glial cells, while 38 target genes are specific to neuronal GRNs and 16 target genes are specific to glial GRNs. We identify 14 targets for Atf4 (Fig. 6H), the majority of which (8) are common to both neurons and glial cells, with only one target gene specific only to neuronal GRNs and five targets specific only to glial GRNs.

## 3 Discussion

We have developed the Inferelator 3.0 software package to scale to match the size of any network inference problem, with no organism-specific requirements that preclude easy application to non-mammalian organisms. Model baselines can be easily established by shuffling labels or generating noised datasets, and cross-validation and scoring on holdout genes is built directly into the pipeline. We believe this is particularly important as evaluation of single-cell network inference tools on real-world problems has lagged behind the development of inference methods themselves. Single-cell data collection has focused on complex higher eukaryotes and left the single-cell network inference field bereft of reliable standards to test against. Recent collection of scRNAseq data from traditional model organisms provides an opportunity to identify successful and unsuccessful strategies for network inference. For example, we find that performance differences between our methods of model selection may be smaller than differences caused by data cleaning and preprocessing. Benchmarking using model organism data should be incorporated in all single-cell method development, as it mitigates cherry-picking from complex network results and can prevent use of flawed performance metrics, which are the only option when no reliable gold standard exists. In organisms without a reliable gold standard, network inference predictions should not be assumed correct and must be validated experimentally (Allaway *et al.*, 2021).

Unlike traditional RNA-seq that effectively measures the average gene expression of large number of cells, scRNAseq can yield individual measurements for many different cell types that are implementing distinct regulatory programs. Learning GRNs from each of these cell types as a separate learning task in a multi-task framework allows cell-type differences to be retained, while still taking advantage of the common regulatory programs. We demonstrate the use of this multi-task approach to simultaneously learn regulatory GRNs for a variety of mouse neuronal cell types from a very large (10^6^) single-cell dataset. This includes learning GRNs for rare cell types; by sharing information between cell types during regression, we are able to learn a core regulatory network while also retaining cell-type-specific interactions. As the GRNs that have been learned for each cell type are sparse and consist of the highest-confidence regulatory edges, they are very amenable to exploration and experimental validation.

A number of limitations remain that impact our ability to accurately predict gene expression and cell states. Most important is a disconnect between the linear modeling that we use to learn GRNs and the non-linear biophysical models that incorporate both transcription and RNA decay. Modeling strategies that more accurately reflect the underlying biology will improve GRN inference directly, and will also allow prediction of useful latent parameters (e.g. RNA half-life) that are experimentally difficult to access. It is also difficult to determine if regulators are activating or repressing specific genes (Kamimoto *et al.*, 2020), complicated further by biological complexity that allows TFs to switch between activation and repression (Papatsenko and Levine, 2008). Improving prediction of the directionality of network edges, and if directionality is stable in different contexts would also be a major advance. Many TFs bind cooperatively as protein complexes, or antagonistically via competitive binding, and explicit modeling of these TF–TF interactions would also improve GRN inference and make novel biological predictions. The modular Inferelator 3.0 framework will allow us to further explore these open problems in regulatory network inference without having to repeatedly reinvent and reimplement existing work. We expect this to be a valuable tool to build biologically relevant GRNs for experimental follow-up, as well as a baseline for further development of computational methods in the network inference field.

## 4 Materials and methods

Additional methods available in Supplementary Methods.

### 4.1 Network inference in *B.subtilis*

Microarray expression data for *B.subtilis* were obtained from NCBI GEO; GSE67023 (Arrieta-Ortiz *et al.*, 2015) (*n* = 268) and GSE27219 (Nicolas *et al.*, 2012) (*n* = 266). GRNs were learned using each expression dataset separately in conjunction with a known prior network (Arrieta-Ortiz *et al.*, 2015) (Supplementary Data S1). Performance was evaluated by AUPR on 10 replicates by holding 20% of the genes in the known prior network out, learning the GRN, and then scoring based on the held-out genes. Baseline shuffled controls were performed by randomly shuffling the labels on the known prior network.

Multi-task network inference uses the same *B.subtilis* prior for both tasks, with 20% of genes held out for scoring. Individual task networks are learned and rank-combined into an aggregate network. Performance was evaluated by AUPR on the held-out genes.

### 4.2 Network inference in *S.cerevisiae*

A large microarray dataset was obtained from NCBI GEO and normalized for a previous publication (Tchourine *et al.*, 2018) (*n* = 2577; 10.5281/zenodo.3247754). In short, these data were preprocessed with limma (Ritchie *et al.*, 2015) and quantile normalized. A second microarray dataset consisting of a large dynamic perturbation screen (Hackett *et al.*, 2020) was obtained from NCBI GEO accession GSE142864 (*n* = 1693). This dataset is the median of three replicate *log*_2_ fold changes of an experimental channel over a control channel (which is the same for all observations). The *log*_2_ fold change is further corrected for each time course by subtracting the *log*_2_ fold change observed at time 0. GRNs were learned using each expression dataset separately in conjunction with a known YEASTRACT prior network (Monteiro *et al.*, 2020; Teixeira *et al.*, 2018) (Supplementary Data S1). Performance was evaluated by AUPR on 10 replicates by holding 20% of the genes in the known prior network out, learning the GRN and then scoring based on the held-out genes in a separate gold standard (Tchourine *et al.*, 2018). Baseline shuffled controls were performed by randomly shuffling the labels on the known prior network.

Multi-task network inference uses the same YEASTRACT prior for both tasks, with 20% of genes held out for scoring. Individual task networks are learned and rank-combined into an aggregate network, which is then evaluated by AUPR on the held-out genes in the separate gold standard.

### 4.3 Single-cell network inference in *S.cerevisiae*

Single-cell expression data for *S.cerevisiae* was obtained from NCBI GEO [GSE125162 (Jackson *et al.*, 2020) and GSE144820 (Jariani *et al.*, 2020)]. Individual cells (*n* = 44 343) are organized into one of 14 groups based on experimental metadata and used as separate tasks in network inference. Genes were filtered such that any gene with fewer than 2217 total counts in all cells (1 count per 20 cells) was removed. Data were used as raw, unmodified counts, were Freeman–Tukey transformed ($\sqrt{x+1}+\sqrt{x}-1$) or were ${\;\text{log}\;}_{2}$ pseudocount transformed (${\;\text{log}\;}_{2}(x+1)$). Data were either not normalized, or depth normalized by scaling so that the sum of all counts for each cell is equal to the median of the sum of counts of all cells. For each set of parameters, network inference is run 10 times, using the YEASTRACT network as prior knowledge with 20% of genes held out for scoring. For noise-only controls, gene expression counts are simulated randomly such that for each gene *i*, $x_{i}∼N(\mu_{x_{i}},\sigma_{x_{i}})$ and the sum for each cell is equal to the sum in the observed data. For shuffled controls, the gene labels on the prior knowledge network are randomly shuffled.

### 4.4 Single-cell network inference in *Mus musculus* neurons

GRNs were learned using AMuSR on log_2_ pseudocount transformed count data for each of 36 cell-type-specific clusters as separate tasks with the appropriate prior knowledge network. An aggregate network was created by rank-summing each cell-type GRN. MCC was calculated for this aggregate network based on a comparison to the union of the three prior knowledge networks, and the confidence score, which maximized MCC was selected as a threshold to determine the size of the final network. Neuron-specific edges were identified by aggregating filtered individual task networks with their respective confidence score to maximize MCC. Each edge that was shared with a glial or vascular network was excluded. The remaining neuron specific edges are interneuron specific, excitatory specific or shared.

## Supplementary Material

btac117_Supplementary_Data
