# Supplementary material for: High-performance single-cell gene regulatory network inference at scale: the Inferelator 3.0
Source: Bioinformatics. 2022 Feb 21;38(9):2519–28. doi: 10.1093/bioinformatics/btac117 (PMC9048651; doi:10.1093/bioinformatics/btac117)
Supplement: btac117_Supplementary_Data [file btac117_supplementary_data.zip › Bioinformatics_GJS_Supplemental_Material.pdf]

High performance single-cell gene regulatory network inference at scale: The Inferelator 3.0

Supplemental Methods and Figures

---

1. Supplemental Methods

1.1. BEELINE Benchmarks

Test data and networks for the BEELINE panel were obtained from Zenodo (DOI: 10.5281/zenodo.3378975). For tests without any prior network information, the Inferelator was provided with expression data and scored against the entire gold-standard network. For tests with prior information, the Inferelator was provided with expression data and half the genes from the gold-standard network as a prior knowledge network. Scoring was performed on genes which were not provided in the prior knowledge network. Network inference was performed on each of expression data sets 10 times, with different random seeds each time. The median AUPR of the 10 network inference runs is reported as the performance for that specific expression data set. AUPR ratios are calculated using the baseline AUPR as defined in the BEELINE benchmarks. Scores for other methods are taken from supplemental data of the previously published BEELINE benchmark.

1.2. Benchmarking CellOracle & Scenic

CellOracle (v 0.7.5) was obtained from GitHub (`https://github.com/morris-lab/CellOracle` commit: cda023a) and installed into a new Anaconda environment. pySCENIC (v0.11.2) was obtained from the python package manager pypi and installed into a new Anaconda environment. A benchmarking module was written for the Inferelator to run CellOracle and pySCENIC from the inferelator workflow. Data loading, crossvalidation, simulation, and scoring functions are identical between all methods. CellOracle was provided the prior knowledge network as a binary dataframe. pySCENIC was provided the prior knowledge network as a ranked-interaction feather database and TF lookup table, in accordance

with the pySCENIC pipeline for generating prior knowledge databases for new organisms. Expression data for pySCENIC was log pseudocount transformed and scaled. Expression data for CellOracle was provided as raw counts, which was then log pseudocount transformed and scaled during CellOracle run.

1.3. Inferelator 3.0 Single-Cell Computational Speed Profiling

144,682 mouse cells from the mouse neuronal subcluster EXC_IT_1 were used with the mouse excitatory neuron prior knowledge network to determine Inferelator 3.0 runtime. To benchmark the python-based multiprocessing engine, the Inferelator was deployed to a single 28-core (Intel® Xeon® E5-2690) node. The Dask implementations of the Inferelator and pySCENIC were deployed to 5 28-core (Intel® Xeon® E5-2690) nodes for a total of 140 cpu cores. Either all 144,682 mouse cells were used, or a subset was randomly selected for each run, and used to learn a single GRN. Runtime was determined by the length of workflow execution, which includes loading data, running all regressions, and producing output files. We were unable to run the full 144k cell data set with pySCENIC due to runtime limitations (with GENIE3) or cryptic memory-related errors (with GRNBOOST2).

1.4. Preprocessing Mus musculus single-cell data

Single-cell expression data from *Mus musculus* brain samples taken at E18 was obtained from 10x genomics (10x Genomics, 2017). SCANPY was used to preprocess and cluster the scRNAseq dataset. Genes present in fewer than 2% of cells were removed. Cells were filtered out when fewer than 1000 genes were detected, the cell had more than 20,000 total gene counts, or the cell had more than 7% of gene counts assigned to mitochondrial transcripts. Transcript counts were then log transformed and normalized and scaled. Cells were assigned to mitotic or post mitotic phase based on cell cycle marker genes using score_genes_cell_cycle (Satija *et al.*, 2015). In order to focus on neuronal cells, all 374,369 mitotic cells were removed. Remaining cells were clustered by Leiden clustering (Resolution = 0.5) using the first 300 principal components of the 2000 most highly variable genes. Broad cell types were assigned to each cluster based on the expression of marker genes Neurod6 for Excitatory neurons, Gad1 for Interneurons, and Apoe for glial cells. Cells from each broad cell type were then re-clustered into clusters based on the 2000 most highly variable genes within the cluster. Specific cell types were assigned to each subcluster based on the expression of marker genes(Di Bella *et al.*, 2020). Ambiguous clusters were discarded, removing

151,765 cells, leaving resulting in 36 specific cell type clusters that consist of 766,402 total cells.

Single-cell ATAC data from *Mus musculus* brain samples taken at E18 was obtained from 10x genomics; datasets are from samples prepared fresh (10x Genomics, 2019c), samples dissociated and cryopreserved (10x Genomics, 2019a), and samples flash-frozen (10x Genomics, 2019b). ChromA (Gabitto *et al.*, 2020) and SnapATAC (Fang *et al.*, 2021) were used to process the scATACseq datasets. Consensus peaks were called on the 3 datasets using ChromA. Each dataset was then run through the SnapATAC pipeline using the consensus peaks. Cells were clustered and labels from the scRNAseq object were transferred to the scATAC data. Cells that did not have an assignment score $\geq .5$ were discarded. Assigned barcodes were split by cell class(EXC, IN or GL). ChromA was run again for each cell class generating 3 sets of cell class specific peaks.

Aggregated chromatin accessibility profiles were used with TRANSFAC v2020.1 motifs and the inferelator-prior (v0.3.0) pipeline to create prior knowledge connectivity matrices between TFs and target genes for excitatory neurons, interneurons, and glial cells. Vascular cells were not present in the scATAC data sufficiently to allow construction of a vascular cell prior with this method, and so vascular cells were assigned the glial prior for network inference.

1.5. Saccharomyces cerevisiae prior knowledge networks

A prior knowledge matrix consists of a signed or unsigned connectivity matrix between regulatory transcription factors (TFs) and target genes. This matrix can be obtained experimentally or by mining regulatory databases. For a TF - gene relationships to be directly causal, the TF must localize to the gene, and gene expression must change in response to perturbations in the TF. However, these criteria do not have to be met at all times. It is reasonable to expect that in many (or most) cell states, a TF may not localize to a target gene, or expression of the gene may not be affected by perturbations in the TF.

Prior knowledge and gold standard networks are selected with these criteria in mind. The YEASTRACT prior knowledge network was obtained from the YEASTRACT database (Teixeira *et al.*, 2018; Monteiro *et al.*, 2020) (`http://www.yeastract.com/`; Downloaded 07/13/2019) which is constructed from published yeast TF localization and gene expression data. This prior knowledge network has 11,486 TF - gene edges from the YEASTRACT database for which evidence exists that the TF localizes to the

target gene, and that the target gene expression changes upon TF perturbation. The yeast gold standard network was constructed in an earlier work (Tchourine *et al.*, 2018) and consists of 1,403 edges, which have multiple pieces of both DNA localization and target gene perturbation evidence.

1.6. TF Motif-Based Connectivity Matrix (inferelator-prior)

Scanning genomic sequence near promoter regions for TF motifs allows for the construction of motif-derived priors which can be further constrained experimentally by incorporating information about chromatin accessibility (Miraldi *et al.*, 2019). We have further refined the generation of prior knowledge matrices with the python inferelator-prior package, which takes as input a gene annotation GTF file, a genomic FASTA file, and a TF motif file, and generates an unsigned connectivity matrix. It has dependencies on the common scientific computing packages NumPy (Harris *et al.*, 2020), SciPy (Virtanen *et al.*, 2020), and scikit-learn (Pedregosa *et al.*, 2011). In addition, it uses the BEDTools kit (Quinlan and Hall, 2010) and associated python interface pybedtools (Dale *et al.*, 2011). The inferelator-prior package (v0.3.0 was used to generate the networks in this manuscript) is available on github (`https://github.com/flatironinstitute/inferelator-prior`) and can be installed through the python package manager pip.

1.6.1. Motif Databases

DNA binding motifs were obtained from published databases. CISBP (Lambert *et al.*, 2019) motifs were obtained from CIS-BP (`http://cisbp.ccbr.utoronto.ca/`; Build 2.00; Downloaded 11/25/2020) and processed into a MEME-format file with the PWMtoMEME module of inferelator-prior. JASPAR (Fornes *et al.*, 2020) motifs were obtained as MEME files from JASPAR (`http://jaspar.genereg.net/`; 8th Release; Downloaded 11/25/2020) . TRANSFAC (Matys *et al.*, 2006) motifs were licensed from geneXplain (`http://genexplain.com/transfac/`; Version 2020.1; Downloaded 09/13/2020) and processed into a MEME-format file with the inferelator-prior motif parsing tools.

1.6.2. Motif Scanning

Genomic regions of interest are identified by locating annotated Transcription Start Sites (TSS) and opening a window that is appropriate for the organism. For microbial species with a compact genome (e.g. yeast), regions of interest are defined as 1000bp upstream and 100bp downstream

of the TSS. For complex eukaryotes with large intergenic regions (e.g. mammals), regions of interest are defined as 50000bp upstream and 2500bp downstream of the TSS. This is further constrained by intersecting the genomic regions of interest with a user-provided BED file, which can be derived from a chromatin accessibility experiment (ATAC-seq) or any other method of identifying chromatin of interest. Within these regions of interest, motif locations are identified using the Find Original Motif Occurrences (FIMO) (Grant *et al.*, 2011) tool from the MEME suite (Bailey *et al.*, 2009), called in parallel on motif chunks to speed up processing. Each motif hit identified by FIMO is then scored for information content (IC) (Kim *et al.*, 2003). IC_i, ranging between 0 and 2 bits, is calculated for each base i in the binding site, where $p_{b,i}$ is the probability of the base b at position i of the motif and $p_{b,bg}$ is the background probability of base b in the genome (Equation 1). Effective information content (EIC) (Equation 2) is the sum of all motif at position i is IC_i penalized with the ℓ_2-norm of the hit IC_i and the consensus motif base at position i, $\mathrm{IC}_{i,\mathrm{consensus}}$.

$$\mathrm{IC}_i = p_{b,i} \log_2 \left(\frac{p_{b,i}}{p_{b,bg}} \right) \tag{1}$$

$$\mathrm{EIC} = \sum_i \mathrm{IC}_i - | \mathrm{IC}_i - \mathrm{IC}_{i,\mathrm{consensus}} |_2^2 \tag{2}$$

1.6.3. Connectivity Matrix

A TF-gene binding score is calculated separately for each TF and gene. Each motif hit for a TF within the region of interest around the gene is identified. Overlapping motif hits are resolved by taking the maximum IC for each overlapping base, penalized with the ℓ_2-norm of differences from the motif consensus sequence. To account for cooperative TF binding effects, any motif hits within 100 bases (25 bases for yeast) are combined, and their EIC scores are summed. The TF-gene binding score is the maximum TF EIC after accounting for overlapping and adjacent TF motifs, and all TF-gene scores are assembled into a Genes x TFs score matrix.

This unfiltered TF-gene score matrix is not sparse as motifs for many TFs are expected to occur often by chance, and TF-gene scores for each TF are not comparable to scores for other TFs as motif position-weight matrices have differing information content. Scores for each TF are clustered using the density-based k-nearest neighbors algorithm DBSCAN (Ester *et al.*, 1996) (MinPts = 0.001 * number of genes, eps = 1). The cluster of TF-gene edges with the highest score values, and any high-score outliers, are retained in the connectivity matrix, and other TF-gene edges are discarded.

1.6.4. CellOracle Connectivity Matrix

CellOracle (Kamimoto *et al.*, 2020) was cloned from github (v0.6.5; `https://github.com/morris-lab/CellOracle`; a0da790). CellOracle was provided a BED file with promoter locations for each gene (200bp upstream of transcription start site to 50bp downstream of transcription start site) and the appropriate MEME file for each motif database. Connectivity matrices were predicted using a false positive rate of 0.02 and a motif score threshold of 6. The inferelator-prior pipeline was run using the same promoter locations and MEME files so that the resulting networks are directly comparable, and the Jaccard index between each network and the YEASTRACT network was calculated. Each motif-based network was used as a prior for inferelator network inference on *Saccharomyces cerevisiae*, with the same 2577 genome-wide expression microarray measurements (Tchourine *et al.*, 2018). 20% of the genes were held out of the prior networks and used for scoring the resulting network inference. The motif-based network files have been included in Supplemental Data 1.

1.7. Network Inference (The Inferelator)

The Inferelator modeling of gene regulatory networks relies on three main modeling assumptions. First, because many transcription factors (TFs) are post transcriptionally controlled and their expression level may not reflect their underlying biological activity, we assume that the activity of a TF can be estimated using expression levels of known targets from prior interactions data (Arrieta-Ortiz *et al.*, 2015; Fu *et al.*, 2011). Second, we assume that gene expression can be modeled as a weighted sum of the activities of TFs (Bonneau *et al.*, 2006; Castro *et al.*, 2019). Finally, we assume that each gene is regulated by a small subset of TFs and regularize the linear model to enforce sparsity.

The Inferelator was initially developed and distributed as an R package (Bonneau *et al.*, 2006; Greenfield *et al.*, 2010; Madar *et al.*, 2010; Greenfield *et al.*, 2013). We have rewritten it as a python package with dependencies on the common scientific computing packages NumPy (Harris *et al.*, 2020), SciPy (Virtanen *et al.*, 2020), pandas (Wes McKinney, 2010), AnnData (Wolf *et al.*, 2018), and scikit-learn (Pedregosa *et al.*, 2011). Scaling is implemented either locally through python or as a distributed computation with the Dask (Rocklin, 2015) parallelization library. The inferelator package (v0.5.6 was used to generate the networks in this manuscript) is available on github (`https://github.com/flatironinstitute/inferelator`) and can be installed through the python package manager pip. The Inferelator takes as input gene expression data and prior information on network

structure, and outputs ranked regulatory hypotheses of the relative strength and direction of each interaction with an associated confidence score.

1.8. Transcription Factor Activity

The expression level of a TF is often not suitable to describe its activity (Schacht *et al.*, 2014). Transcription factor activity (TFA) is an estimate of the latent activity of a TF that is inducing or repressing transcription of its targets in a sample. A gene expression dataset ($\mathbf{X}$) is a Samples x Genes matrix where $X_{i,j}$ is the observed mRNA expression level ($i \in$ Samples and $j \in$ Genes), measured either by microarray, RNA-seq, or single cell RNA sequencing (scRNA-seq).

$$X_{i,j} = \sum_k A_{i,k} P_{k,j} \tag{3}$$

We estimate TFA by solving (Equation 3) for activity ($A_{i,k}$), where $k \in$ TFs, and $\mathbf{P}$ is a TFs x Genes prior connectivity matrix. $P_{k,j}$ is non-zero if gene j is regulated by TF k and 0 if it is not. In matrix notation, $\mathbf{X} = \mathbf{AP}$, and $\hat{\mathbf{A}}$ is estimated by minimizing $\| \hat{\mathbf{A}}\mathbf{P} - \mathbf{X} \|_2^2$. This is calculated by the pseudoinverse $\mathbf{P}^\dagger$ and solving $\hat{\mathbf{A}} = \mathbf{X}\mathbf{P}^\dagger$. The resulting $\hat{\mathbf{A}}$ is a Samples x TF activities matrix where $\hat{A}_{i,k}$ is the estimated latent TFA for sample i and TF k. In cases where all values in $\mathbf{P}$ for a TF are 0, that TF is removed from $\mathbf{P}$ and the expression $\mathbf{X}$ of that TF is used in place of activity.

1.9. Inferelator Network Inference

Linear models (Equation 4) are separately constructed for each gene j.

$$X_i = \sum_k \hat{A}_{i,k} \beta_k \tag{4}$$

In addition to the model selection methods described here, we have implemented a module which takes any scikit-learn regression object (for example, elastic net (Zou and Hastie, 2005)). Model selection and regularization techniques are applied to enforce the biological property of sparsity. If the coefficient $\beta_{j,k}$ is non-zero, it is evidence for a regulatory relationship between TF k and gene j.

$$S_{j,k} = 1 - \frac{\sigma^2_{allTFs}}{\sigma^2_{TF_k leaveout}} \tag{5}$$

For each gene j, the amount of variance explained by each regulatory TF k is calculated as the ratio between the variance of the residuals in the full

model and the variance of the residuals when the linear model is refit by ordinary least squares (OLS) and k is left out (Equation 5).

In order to mitigate the effect of outliers and sampling error, model selection is repeated multiple times using input expression data $\mathbf{X}$ that has been bootstrapped (resampled with replacement). Predicted TF-gene interactions are ranked for each bootstrap by amount of variance explained and then rank-combined into a unified network prediction. Confidence scores are assigned based on the combined rank for each interaction, and the overall network is compared to a gold standard and performance is evaluated by area under the precision-recall curve.

The effects of setting hyperparameters can be tested by cross-validation on the prior and gold standard networks. This strategy holds out a subset of genes (rows) from the prior knowledge network $\mathbf{P}$. Network inference performance is then evaluated on only those held-out genes, using the gold standard network.

1.9.1. Model Selection: Bayesian Best Subset Regression

Bayesian Best Subset Regression (BBSR) is a model selection method described in detail in (Greenfield *et al.*, 2013). Initial feature selection for this method is necessary as best subset regression on all possible combinations of hundreds of TF features is computationally intractible. We therefore select ten TF features with the highest context likelihood of relatedness between expression of each gene and activity of each TF. This method is described in detail in (Madar *et al.*, 2010).

First, gene expression and TF activity are discretized into equal-width bins (n=10) and mutual information is calculated based on their discrete probability distributions (Equation 6) to create a mutual information matrix $\mathbf{M^{dyn}}$.

$$M_{j,k}^{dyn} = p(X_j, \hat{A}_k) \log \frac{p(X_j, \hat{A}_k)}{p(X_j)p(\hat{A}_k)} \tag{6}$$

$$M_{k_1,k_2}^{stat} = p(\hat{A}_{k_1}, \hat{A}_{k_2}) \log \frac{p(\hat{A}_{k_1}, \hat{A}_{k_2})}{p(\hat{A}_{k_1})p(\hat{A}_{k_2})} \tag{7}$$

Mutual information is also calculated between activity of each TF (Equation 7) to create a mutual information matrix $\mathbf{M^{stat}}$.

$$z_{j,k}^{dyn} = \frac{Mj,k^{dyn} - \sum_j \frac{Mj,k^{dyn}}{n_i}}{\sigma_k^{dyn}} \tag{8}$$

$$z_{j,k}^{stat} = \frac{Mj, k^{dyn} - \sum_j \frac{Mj,k^{stat}}{n_i}}{\sigma_k^{stat}} \tag{9}$$

$$z_{j,k}^{mixed} = \sqrt{(z_{j,k}^{dyn})^2 + (z_{j,k}^{stat})^2} \tag{10}$$

A mixed context likelihood of relatedness score is then calculated as a pseudo-zscore by calculating $\mathbf{Z^{dyn}}$ (Equation 8) and $\mathbf{Z^{stat}}$ (Equation 9). Any values less than 0 in $\mathbf{Z^{dyn}}$ or $\mathbf{Z^{stat}}$ are set to 0, and then they are combined into a mixed context likelihood of relatedness matrix $\mathbf{Z^{mixed}}$ (Equation 10). For each gene j, the 10 TFs with the highest mixed context likelihood of relatedness values are selected for regression.

For best subset regression, a linear model is fit with OLS for every combination of the selected predictor variables.

$$\rho(\beta, \sigma^2 | X_j) = \rho(\beta | X_j, \sigma^2) \rho(\sigma^2 | X_i) \tag{11}$$

$$\rho(\sigma^2 | X_i) \propto IG(\frac{n}{2}, \frac{SSR}{2} + \frac{(\beta_0 - \beta_{\text{OLS}})\mathbf{GX'XG}(\beta_0 - \beta_{\text{OLS}})}{2}) \tag{12}$$

We define β_0 as our null prior for the model parameters (zeros), β_{OLS} as the model coefficients from OLS, SSR as the sum of squared residuals, and $\mathbf{G}$ as a g-prior diagonal matrix where the diagonal values represent a weight for each predictor variable. g-prior weights in $\mathbf{G}$ close to 0 favor β values close to β_0. Large g-prior weights favor β values close to β_{OLS}. By default, we select g-prior weights of 1 for all predictor variables. From the joint posterior distribution (Equation 11) we can calculate the marginal posterior distribution of σ^2 (Equation 12), where IG is the inverse gamma distribution. The Bayesian information criterion (BIC) is calculated for each model, where n is the number of observations and k is the number of predictors (Equation 13).

$$BIC = n \ln{(\sigma^2)} - k \ln{(n)} \tag{13}$$

$$E[\sigma^2] = \frac{\frac{SSR}{2} + \frac{(\beta_0 - \beta_{\text{OLS}})\mathbf{GX'XG}(\beta_0 - \beta_{\text{OLS}})}{2}}{\frac{n}{2} - 1} \tag{14}$$

$$E[BIC] = n(\ln{(\frac{SSR}{2} + \frac{(\beta_0 - \beta_{\text{OLS}})\mathbf{GX'XG}(\beta_0 - \beta_{\text{OLS}})}{2})} - Digamma(\frac{n}{2})) - k \ln(n) \tag{15}$$

We calculate the expected posterior distribution of σ^2 (Equation 14) for each subset of predictors, and use it to determine the model BIC (Equation 15). We then select the model with the smallest $E[BIC]$. The predictors in the selected subset model for gene j are TFs which regulate its expression.

1.9.2. Model Selection: StARS-LASSO

Least absolute shrinkage and selection operator (LASSO) (Zou, 2006) combined with the Stability Approach to Regularization Selection (StARS) (Liu *et al.*, 2010) is a model selection method described in detail in (Miraldi *et al.*, 2019). In short, the StARS-LASSO approach is to select the optimal λ parameter for (Equation 16). N random subsamples of X and $\hat{A}$ without replacement subnetworks $S_{n,\lambda}$ are defined as the non-zero coefficients $\beta_{n,\lambda}$ after LASSO regression. Initially, λ is set large, so that each subnetwork S_n is highly sparse, and is then decreased, resulting in increasingly dense networks. Edge instability is calculated as the fraction of times subnetworks disagree about the presence of an network edge. As λ decreases, the subnetworks are expected to have increasing edge instability initially and then decreasing edge instability as λ approaches 0, as (Equation 16) reduces to OLS and each subnetwork becomes dense.

$$\min_{\beta} \frac{1}{2n} |X - \hat{A}\beta|_2^2 - \lambda|\beta|_1 \tag{16}$$

We choose the largest value of λ such that the edge instability is less than 0.05, which is interpretable as all subnetworks share $> 95\%$ of edges. This selection represents a balance between increasing the network size and minimizing the instability that occurs when data is sampled.

1.10. Multiple Task Network Inference

We separate biological samples which represent different states into separate tasks, learn networks from these tasks, and then combine task-specific networks into an ensemble network. One method of solving these states is to sequentially apply a single-task method for network inference (i.e. 1.9.1 or 1.9.2). The networks generated for each task are then rank-combined into a unified network. The Adaptive Multiple Sparse Regression (AMuSR) method, described in detail in (Castro *et al.*, 2019), uses a multi-task learning framework, where each task is solved together.

$$\arg\min_{B,S_d} \frac{1}{2n} \|X_{d,i} - (S_d + B)\hat{A}_d\|_2^2 + \lambda_s \|S_d\|_{1,1} + \lambda_b \|B\|_{1,\infty} \tag{17}$$

$$\hat{W}_d = \hat{B} + \hat{S}_d \tag{18}$$

In (Equation 17), B is a block-sparse weight matrix in which the weights for any feature are the same across all tasks. S_d is a sparse weight matrix for task d, allowing weights for features to vary between tasks. The combination W_d of B and S_d (Equation 18) are model weights representing regulatory

interactions between TFs and genes for task d. In short, this method uses adaptive penalties to favor regulatory interactions shared across multiple tasks in B, while recognizing dataset specific interactions in S_d. Model hyperparameters λ_s and λ_b are identified by grid search, selecting the model that minimizes the extended Bayesian Information Criterion (eBIC) (Equation 19), where D is the number of task datasets, and for dataset d, n_d is the number of observations, $X_i^{(d)}$ is gene expression for gene i, $\hat{A}^{(d)}$ is TF activity estimates, $W_{*,d}$ is model weights, k_d is the number of non-zero predictors, and p_d is the total number of predictors. For this work, we choose to set the eBIC paramater γ to 1.

$$eBIC = \frac{1}{D} \sum n_d \ln \frac{1}{n_d} \|X_i^{(d)} - \hat{A}^{(d)T} W_{*,d}\|_2^2 + k_d \ln n_d + 2\gamma \ln \binom{p_d}{k_d} \quad (19)$$

1.11. Network Performance Metrics

Prior work has used the area under the Precision (Equation 20) - Recall (Equation 21) curve to determine performance, by comparing to some known, gold-standard network. Here we add two metrics; Matthews correlation coefficient (Matthews, 1975) (MCC) (Equation 22) and F1 score (Equation 23). MCC can be calculated directly from the confusion matrix True Positive (TP), False Positive (FP), True Negative (TN), and False Negative (FN) values.

$$Precision = \frac{TP}{TP + FP} \quad (20)$$

$$Recall = \frac{TP}{TP + FN} \quad (21)$$

$$MCC = \frac{TP * TN - FP * FN}{\sqrt{(TP + FP)(TP + FN)(TN + FP)(TN + FN)}} \quad (22)$$

$$F1 = 2 * \frac{Precision * Recall}{Precision + Recall} \quad (23)$$

We compute an MCC and F1 score for each cutoff along ranked interactions in order to generate MCC and F1 scores for all possible networks in growing ranked order. The maximum MCC along ranked interactions gives the subnetwork that has maximum similarity to the comparison network, accounting for TP, FP, TN, and FN. The maximum F1 along ranked interactions gives the subnetwork that has maximum similarity to the comparison network accounting for TP, FP, and FN.

1.12. Visualization

Figures were generated with R (R Core Team, 2020) and the common ggplot2 (Wickham, 2016), umap (McInnes *et al.*, 2018), and tidyverse packages (Wickham *et al.*, 2019). Additional figures were generated with python using scanpy (Wolf *et al.*, 2018), matplotlib (Hunter, 2007), and seaborn (Waskom, 2021). Network diagrams were created with the python package jp_gene_viz (Watters, 2019). Schematic figures were created in Adobe Illustrator, and other figures were adjusted in Illustrator to improve panelling and layout.

Availability of Data and Materials

The datasets supporting the conclusions of this article are available in the NCBI GEO repository with accession IDs: GSE125162, GSE144820, GSE67023, GSE27219, GSE142864. A large number of GEO records were compiled and normalized in a previous work Tchourine *et al.* (2018) into a combined dataset which is available on Zenodo (DOI: 10.5281/zenodo.3247754). The scRNAseq expression matrix, metadata, prior knowledge network, and gold standard network for the yeast network inference benchmarking is available on Zenodo (DOI: 10.5281/zenodo.5272314). Single-cell mouse datasets are publicly available from 10x genomics 10x Genomics (2017, 2019c,a,b) under a Creative Commons Attribution (CC-BY 4.0) license. Software packages developed for this article are available on github (`https://github.com/flatironinstitute/inferelator` and `https://github.com/flatironinstitute/inferelator-prior`) and have been released as python packages through PyPi (`https://pypi.org/project/inferelator/` and `https://pypi.org/project/inferelator-prior/`). Specific analysis scripts for this work have been included in Supplemental Data 1.

Author's contributions

CSG contributed to Methodology, Software, Validation, Formal Analysis, Writing – Original Draft Preparation, and Visualization. CJ and GS contributed to Conceptualization, Methodology, Software, Validation, Investigation, Resources, Data Curation, Formal Analysis, Writing – Original Draft Preparation, and Visualization. AS contributed to Validation, Data Curation, Formal Analysis, and Visualization. AW contributed to Software and Visualization. AT contributed to Software, Writing – Original Draft Preparation, and Formal Analysis. DC and KT contributed to Software, Data Curation, and Conceptualization. NDV, NC, RY, and TH contributed

to Software. DG contributed to Supervision, Project Administration, and Funding Acquisition. EM contributed to Conceptualization, Writing – Original Draft Preparation, and Software. RB contributed to Conceptualization, Writing – Original Draft Preparation, Supervision, Project Administration, and Funding Acquisition.

Additional Files

- Supplemental Data 1 is a .tar.gz file containing the prior knowledge networks used in this work, the gold standard networks used in this work, and the python scripts used to generate the learned networks in this work
- Supplemental Data 2 is a .tar.gz file containing the mouse E18 neuronal network learned in Figure 6 of this work
- Supplemental Table 1 is a .tsv file containing the crossvalidation performance results from Figure 2
- Supplemental Table 2 is a .tsv file containing the crossvalidation performance results from Figure 3
- Supplemental Table 3 is a .tsv file containing the crossvalidation performance results from Figure 4B-D
- Supplemental Table 4 is a .tsv file containing the crossvalidation performance results from Figure 4G
- Supplemental Table 5 is a .tsv file containing the crossvalidation performance results from Supplemental Figure 5A
- Supplemental Table 6 is a .tsv file containing the crossvalidation performance results from Figure 4H
- Supplemental Table 7 is a .tsv file containing the crossvalidation performance results from Supplemental Figure 3

2. Supplemental References

10x Genomics (2017). 1.3 million brain cells from e18 mice. https://support.10xgenomics.com/single-cell-gene-expression/datasets/1.3.0/1M_neurons.

10x Genomics (2019a). Dissociated and cryopreserved cortex, hippocampus, and ventricular zone cells from embryonic mouse brain (e18). https://support.10xgenomics.com/single-cell-atac/datasets/1.2.0/atac_v1_E18_brain_cryo_5k.

10x Genomics (2019b). Flash frozen cortex, hippocampus, and ventricular zone from embryonic mouse brain (e18). https://support.10xgenomics.com/single-cell-atac/datasets/1.2.0/atac_v1_E18_brain_flash_5k.

10x Genomics (2019c). Fresh cortex, hippocampus, and ventricular zone from embryonic mouse brain (e18). https://support.10xgenomics.com/single-cell-atac/datasets/1.2.0/atac_v1_E18_brain_fresh_5k.

Arrieta-Ortiz, M.L. et al (2015). An experimentally supported model of the bacillus subtilis global transcriptional regulatory network. *Mol. Syst. Biol.*, **11**(11), 839.

Bailey, T.L. et al (2009). MEME SUITE: tools for motif discovery and searching. *Nucleic Acids Res.*, **37**(Web Server issue), W202–8.

Bonneau, R. et al (2006). The inferelator: an algorithm for learning parsimonious regulatory networks from systems-biology data sets de novo. *Genome Biol.*, **7**, R36.

Castro, D.M. et al (2019). Multi-study inference of regulatory networks for more accurate models of gene regulation. *PLoS Comput. Biol.*, **15**(1), e1006591.

Dale, R.K. et al (2011). Pybedtools: a flexible python library for manipulating genomic datasets and annotations. *Bioinformatics*, **27**(24), 3423–3424.

Di Bella, D.J. et al (2020). Molecular logic of cellular diversification in the mammalian cerebral cortex.

Ester, M. et al (1996). A density-based algorithm for discovering clusters in large spatial databases with noise. In *Proceedings of the Second International Conference on Knowledge Discovery and Data Mining*, KDD'96, pages 226–231. AAAI Press.

Fang, R. et al (2021). Comprehensive analysis of single cell atac-seq data with snapatac. *Nature Communications*, **12**(1), 1337.

Fornes, O. et al (2020). JASPAR 2020: update of the open-access database of transcription factor binding profiles. *Nucleic Acids Res.*, **48**(D1), D87–D92.

Fu, Y. et al (2011). Reconstructing genome-wide regulatory network of e. coli using transcriptome data and predicted transcription factor activities. *BMC Bioinformatics*, **12**, 233.

Gabitto, M.I. et al (2020). Characterizing chromatin landscape from aggregate and single-cell genomic assays using flexible duration modeling. *Nature Communications*, **11**(1), 747.

Grant, C.E. et al (2011). FIMO: scanning for occurrences of a given motif. *Bioinformatics*, **27**(7), 1017–1018.

Greenfield, A. et al (2010). DREAM4: Combining genetic and dynamic information to identify biological networks and dynamical models. *PLoS One*, **5**(10), e13397.

Greenfield, A. et al (2013). Robust data-driven incorporation of prior knowledge into the inference of dynamic regulatory networks. *Bioinformatics*, **29**(8), 1060–1067.

Harris, C.R. et al (2020). Array programming with NumPy. *Nature*, **585**(7825), 357–362.

Hunter, J.D. (2007). Matplotlib: A 2D graphics environment. *Computing in Science Engineering*, **9**(3), 90–95.

Kamimoto, K. et al (2020). CellOracle: Dissecting cell identity via network inference and in silico gene perturbation.

Kim, J.T. et al (2003). Bioinformatic principles underlying the information content of transcription factor binding sites. *J. Theor. Biol.*, **220**(4), 529–544.

Lambert, S.A. et al (2019). Similarity regression predicts evolution of transcription factor sequence specificity. *Nat. Genet.*, **51**(6), 981–989.

Liu, H. et al (2010). Stability approach to regularization selection (StARS) for high dimensional graphical models. *arXiv*.

Madar, A. et al (2010). DREAM3: Network inference using dynamic context likelihood of relatedness and the inferelator. *PLoS One*, **5**(3), e9803.

Matthews, B.W. (1975). Comparison of the predicted and observed secondary structure of T4 phage lysozyme. *Biochim. Biophys. Acta*, **405**(2), 442–451.

Matys, V. et al (2006). TRANSFAC and its module TRANSCompel: transcriptional gene regulation in eukaryotes. *Nucleic Acids Res.*, **34**(Database issue), D108–10.

McInnes, L. et al (2018). UMAP: Uniform manifold approximation and projection for dimension reduction. *arXiv:1802.03426 [cs, stat]*.

Miraldi, E.R. et al (2019). Leveraging chromatin accessibility for transcriptional regulatory network inference in T helper 17 cells. *Genome Res.*

Monteiro, P.T. et al (2020). YEASTRACT+: a portal for cross-species comparative genomics of transcription regulation in yeasts. *Nucleic Acids Res.*, **48**(D1), D642–D649.

Pedregosa, F. et al (2011). Scikit-learn: Machine learning in python. *J. Mach. Learn. Res.*, **12**(Oct), 2825–2830.

Quinlan, A.R. and Hall, I.M. (2010). BEDTools: a flexible suite of utilities for comparing genomic features. *Bioinformatics*, **26**(6), 841–842.

R Core Team (2020). *R: A Language and Environment for Statistical Computing*. R Foundation for Statistical Computing, Vienna, Austria.

Rocklin, M. (2015). Dask: Parallel computation with blocked algorithms and task scheduling. In *Proceedings of the 14th Python in Science Conference*, Proceedings of the Python in Science Conference, pages 126–132. SciPy.

Satija, R. et al (2015). Spatial reconstruction of single-cell gene expression data. *Nat. Biotechnol.*, **33**(5), 495–502.

Schacht, T. et al (2014). Estimating the activity of transcription factors by the effect on their target genes. *Bioinformatics*, **30**(17), i401–7.

Tchourine, K. et al (2018). Condition-Specific modeling of biophysical parameters advances inference of regulatory networks. *Cell Rep.*, **23**(2), 376–388.

Teixeira, M.C. et al (2018). YEASTRACT: an upgraded database for the analysis of transcription regulatory networks in saccharomyces cerevisiae. *Nucleic Acids Res.*, **46**(D1), D348–D353.

Virtanen, P. et al (2020). SciPy 1.0: fundamental algorithms for scientific computing in python. *Nat. Methods*, **17**.

Waskom, M.L. (2021). seaborn: statistical data visualization. *Journal of Open Source Software*, **6**(60), 3021.

Watters, A. (2019). jp_gene_viz. `https://github.com/simonsfoundation/jp_gene_viz`.

Wes McKinney (2010). Data Structures for Statistical Computing in Python. In Stéfan van der Walt and Jarrod Millman, editors, *Proceedings of the 9th Python in Science Conference*, pages 56 – 61.

Wickham, H. (2016). *ggplot2: Elegant Graphics for Data Analysis*. Springer-Verlag New York.

Wickham, H. et al (2019). Welcome to the tidyverse. *J. Open Source Softw.*, **4**(43), 1686.

Wolf, F.A. et al (2018). SCANPY: large-scale single-cell gene expression data analysis. *Genome Biol.*, **19**(1), 15.

Zou, H. (2006). The adaptive lasso and its oracle properties. *J. Am. Stat. Assoc.*, **101**(476), 1418–1429.

Zou, H. and Hastie, T. (2005). Regularization and variable selection via the elastic net. *J. R. Stat. Soc. Series B Stat. Methodol.*, **67**(2), 301–320.

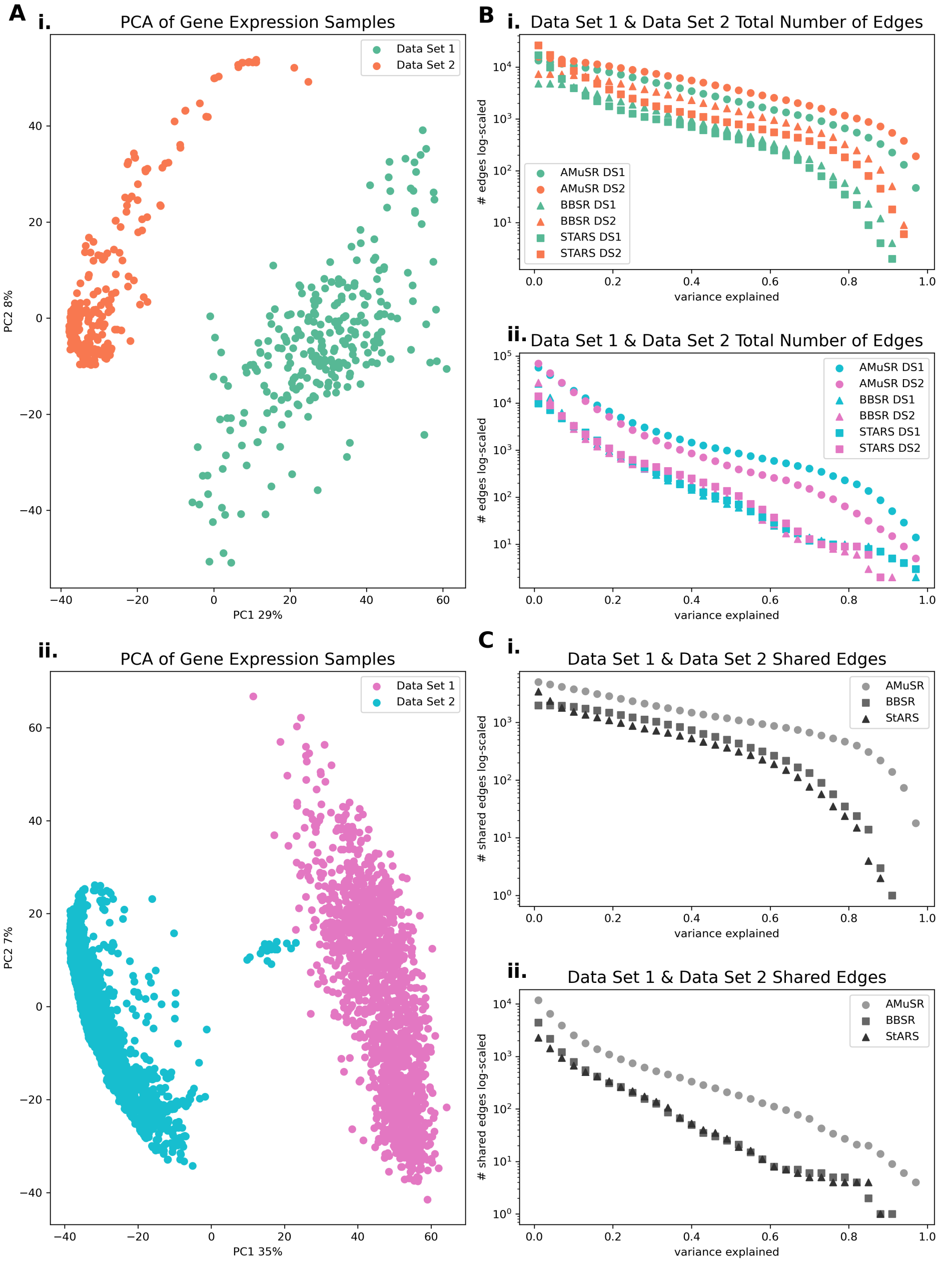

Supplemental Figure 1: Learning *Bacillus subtilis* and *Saccharomyces cerevisiae* networks by tasks. (**A**) PCA depicts batch effects between datasets for both (i) *Bacillus subtilis* and (ii) *Saccharomyces cerevisiae*. Learning networks by treating the independently collected datasets as separate tasks allows for sharing regulatory commonalities while respecting experimental variance. (**B**) The number of shared edges between the two datasets, for both model organisms (i) and (ii), shows a high number of overlapping edges. Edges are ranked by their corresponding variance explained for each of the three different model selection approaches: AMuSR, BBSR, and StARS-LASSO. (**C**) Across the three different model selection approaches, AMuSR learns the highest number of overlapping edges between the respective datasets for model organisms (**i**) and (**ii**).

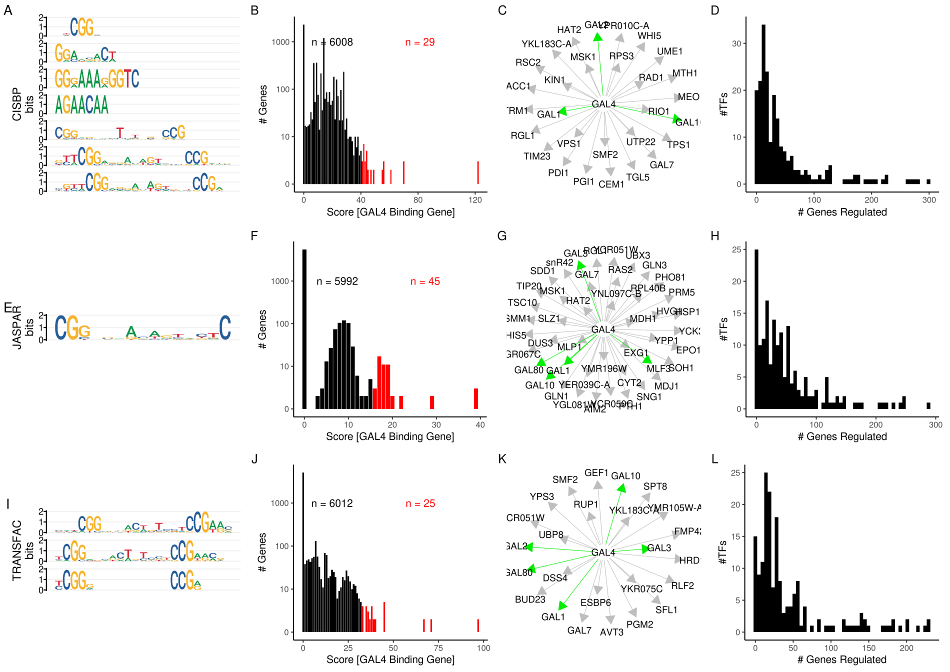

Supplemental Figure 2: Network construction using TF motifs in Saccharomyces cerevisiae. (**A**) Motifs annotated for GAL4 in the CIS-BP motif database. (**B**) Histogram of scores linking GAL4 to target genes. Genes in black have been omitted from the final connectivity matrix, and genes in red have been included. (**C**) Network connecting GAL4 and target genes. Green edges are present in the YEASTRACT database. (**D**) Histogram of out degree for each TF in the complete network. (**E-H**) Network analysis as A-D for the JASPAR motif database. (**I-L**) Network analysis as A-D for the TRANSFAC PRO motif database.

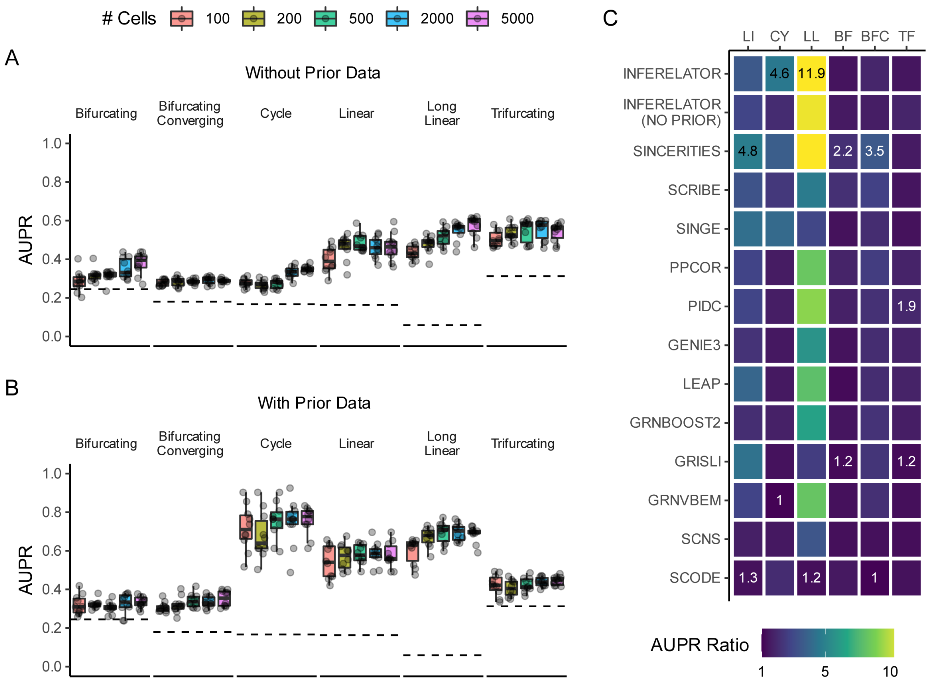

Supplemental Figure 3: Inferelator performance on BEELINE simulated network data. (**A**) Network inference performance of the Inferelator with BBSR model selection as measured by AUPR against the ground truth with no prior network information provided. Dashed lines are the expected baseline of a random predictor. (**B**) Network inference performance of the Inferelator with BBSR model selection as measured by AUPR against half of the ground truth with the other half of the ground truth provided as prior network information. Each point is the median performance of 10 differently-seeded splits. (**C**) Comparison of the AUPR ratio over the baseline for the Inferelator to each of the network inference methods used in the original BEELINE benchmark.

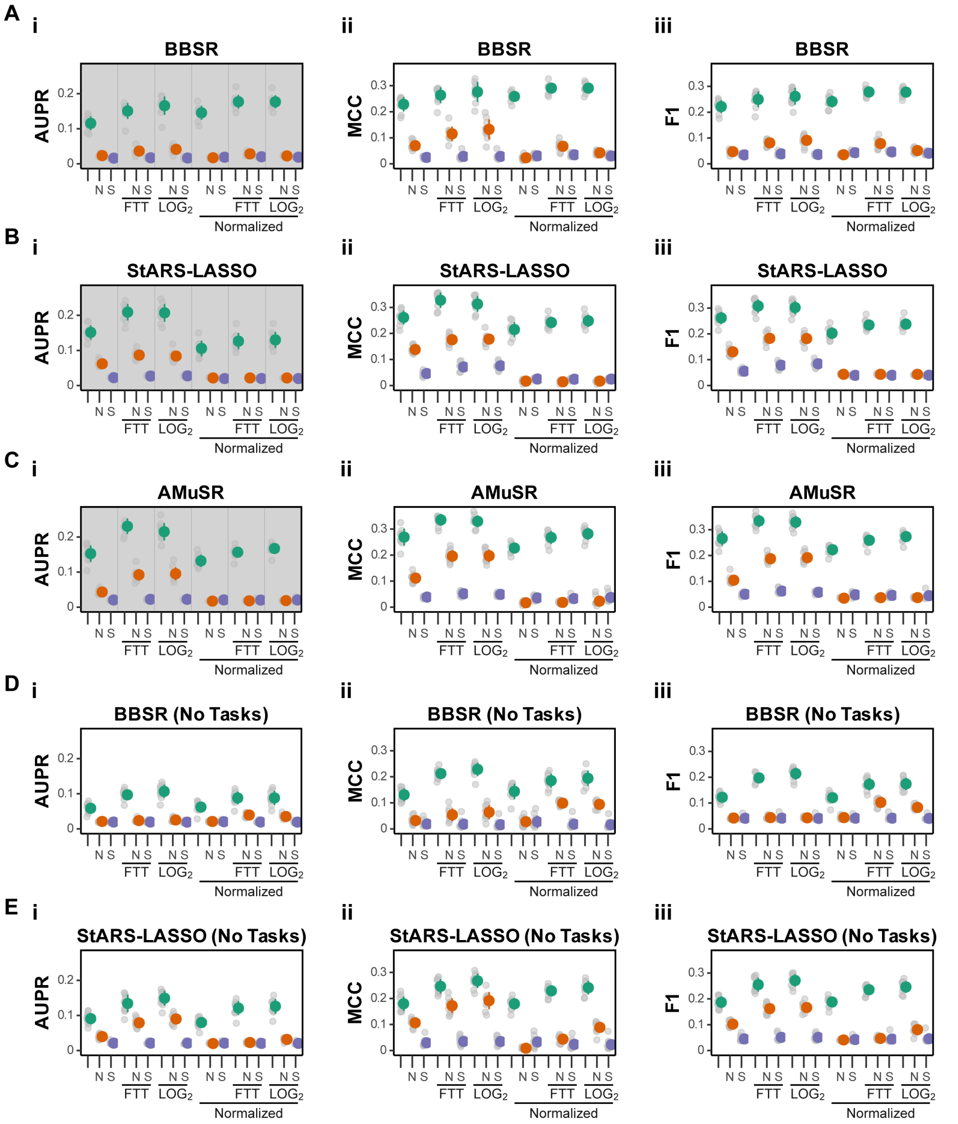

Supplemental Figure 4: Extended single-cell yeast network performance metrics as measured by (**i**) AUPR, (**ii**) Matthews Correlation Coefficient (MCC), and (**iii**) F1 score. Each gray dot represents performance of one network inference run. Colored dots represent the mean and standard deviation. (**A**) Single-cell yeast network inference performance of BBSR model selection Plots with a gray background are the same plots as used in main-text Figure 4. (**B**) Performance of StARS-LASSO model selection. (**C**) Performance of AMuSR model selection. (**D**) Performance of BBSR model selection where all cells are used without splitting into multiple tasks. (**E**) Performance of StARS-LASSO model selection where all cells are used without splitting into multiple tasks.

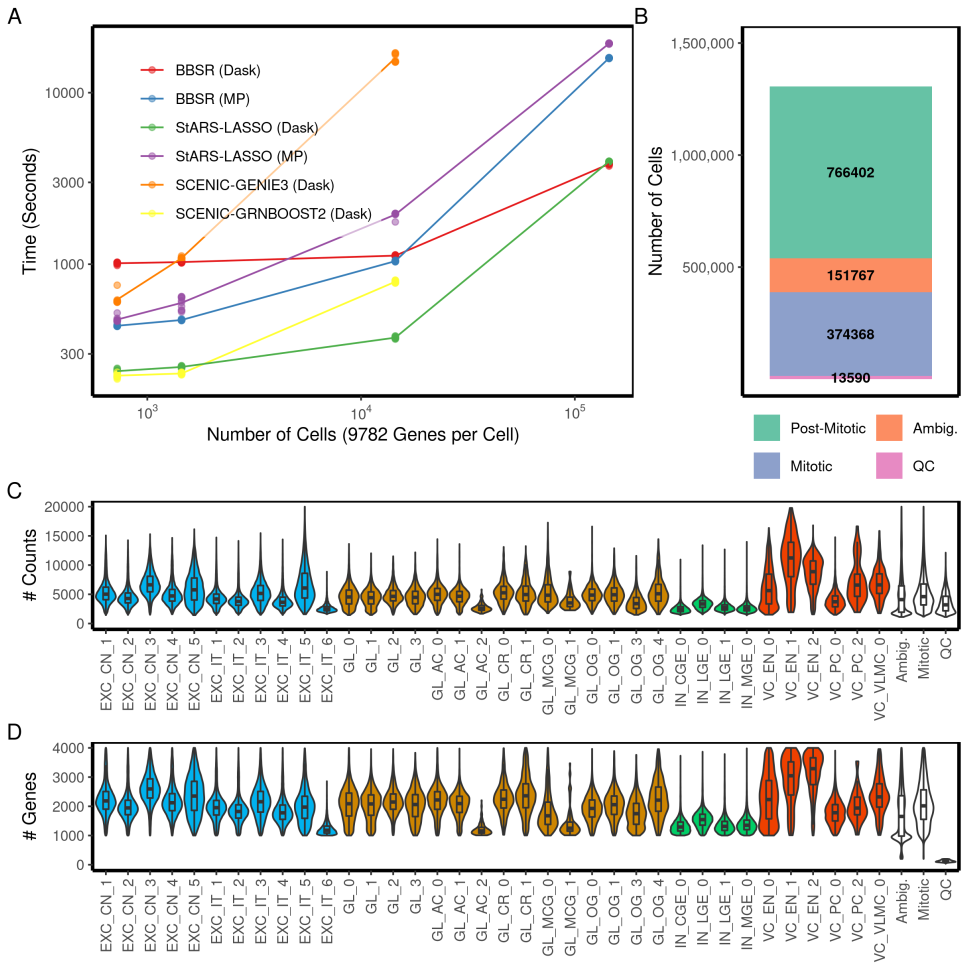

Supplemental Figure 5: (**A**) Computational performance as measured by runtime in seconds using the Dask engine (140 cpu cores) for the Inferelator 3.0 (BBSR or StARS-LASSO), and for SCENIC (GENIE3 or GRNBOOST2). Performance is also measured for the Inferelator 3.0 or using the python-based multprocessing (MP) engine (28 cpu cores). Expression data is sampled from 144,000 mouse cells and 9,782 genes are modeled for network inference. Runtime is shown for 10 replicate runs for each quantity of cells. (**B**) Number of cells removed during preprocessing for Quality Control (QC), as Mitotic, and as Ambiguous by neuronal marker. Post-mitotic, non-ambiguous cells are retained and clustered. (**C**) Number of single-cell counts per cell in each of 36 cell type-specific groups, and in the groups removed during preprocessing. (**D**) Number of genes per cell in each of 36 cell type-specific groups, and in the groups removed during preprocessing

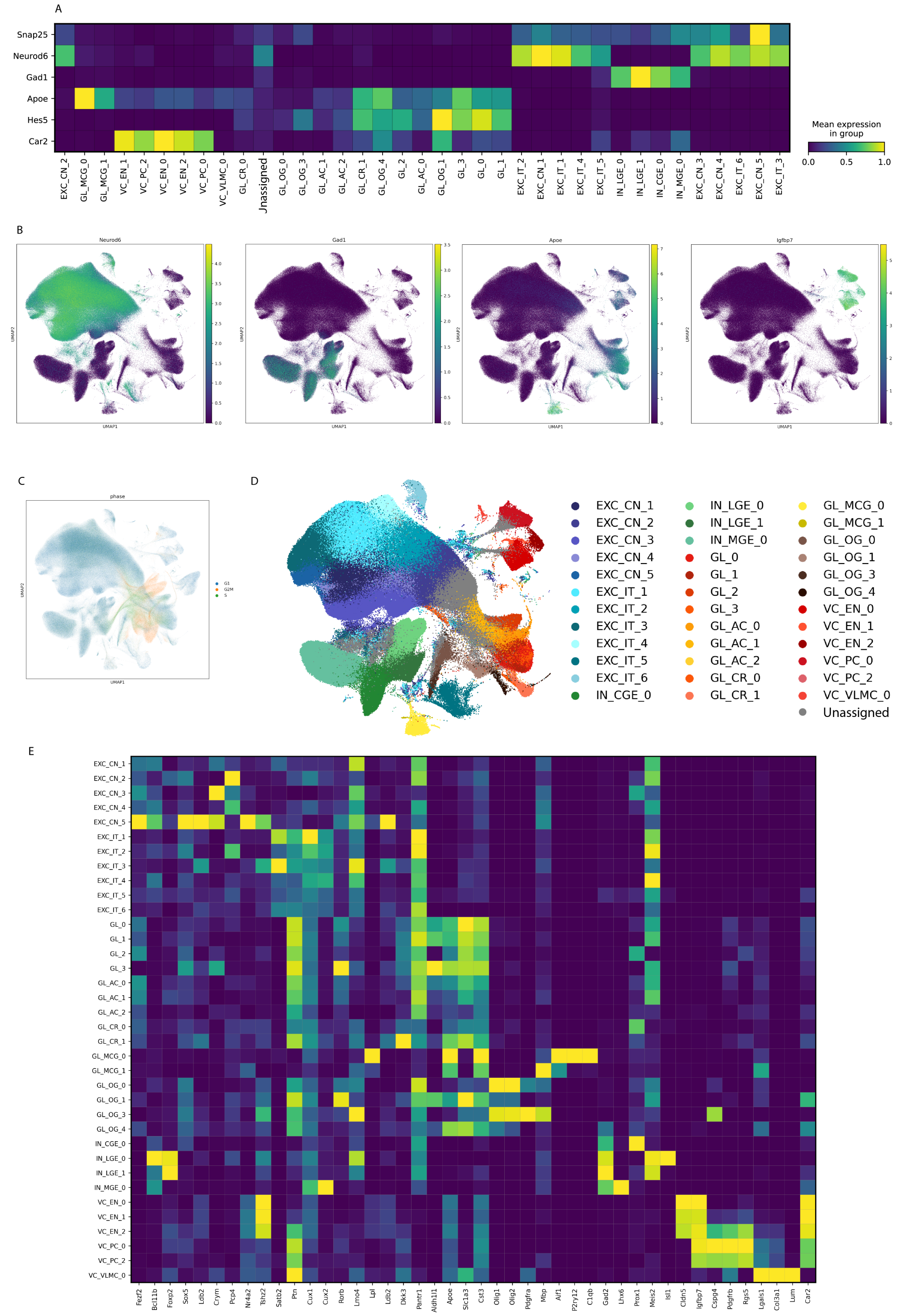

Supplemental Figure 6: (**A**) Cell class marker expression for each annotated subcluster in mouse single-cell brain data. (**B**) UMAP of 766,402 mouse brain cells colored by cell class marker expression. (**C**) UMAP of 1.3M mouse brain cells colored by the assigned cell cycle phase. (**D**) UMAP of 766,402 mouse brain cells colored by 36 assigned subcluster. (**E**) Cell type marker expression by assigned subcluster.

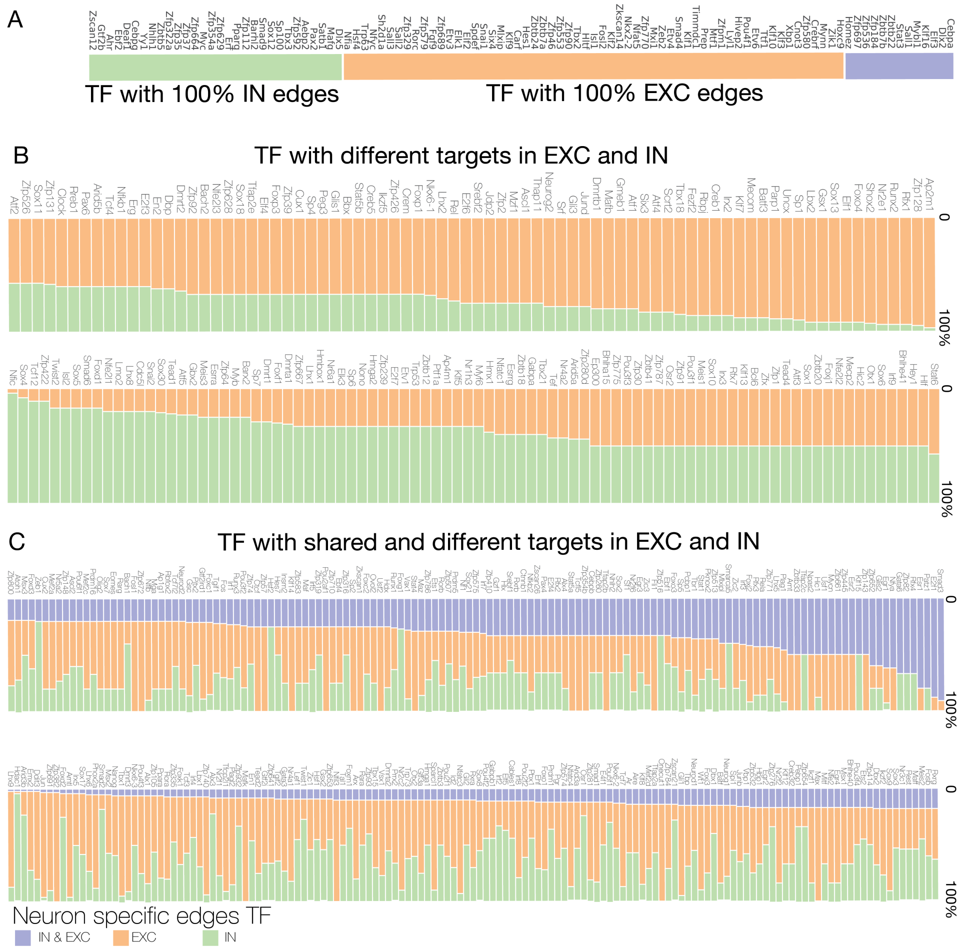

Supplemental Figure 7: (**A**) List of TFs that have identical target genes in GRNs for both Excitatory neurons (EXC) and Interneurons (IN), that have only target genes in Excitatory neurons, and that have only target genes in Interneurons. (**B**) List of TFs that have no shared target genes in GRNs for Excitatory neurons and in GRNs for interneurons. (**C**) TFs that have some shared target genes in GRNs for Excitatory neurons and interneurons, but also have some target genes specific to Excitatory neurons or interneurons.
