# Supplementary material for: High-performance single-cell gene regulatory network inference at scale: the Inferelator 3.0
Source: Bioinformatics. 2022 Feb 21;38(9):2519–28. doi: 10.1093/bioinformatics/btac117 (PMC9048651; doi:10.1093/bioinformatics/btac117)
Supplement: btac117_Supplementary_Data [file btac117_supplementary_data.zip › Bioinformatics_GJS_Supplemental_Data_2.tar.gz!/GL_1/combined_metrics.pdf]

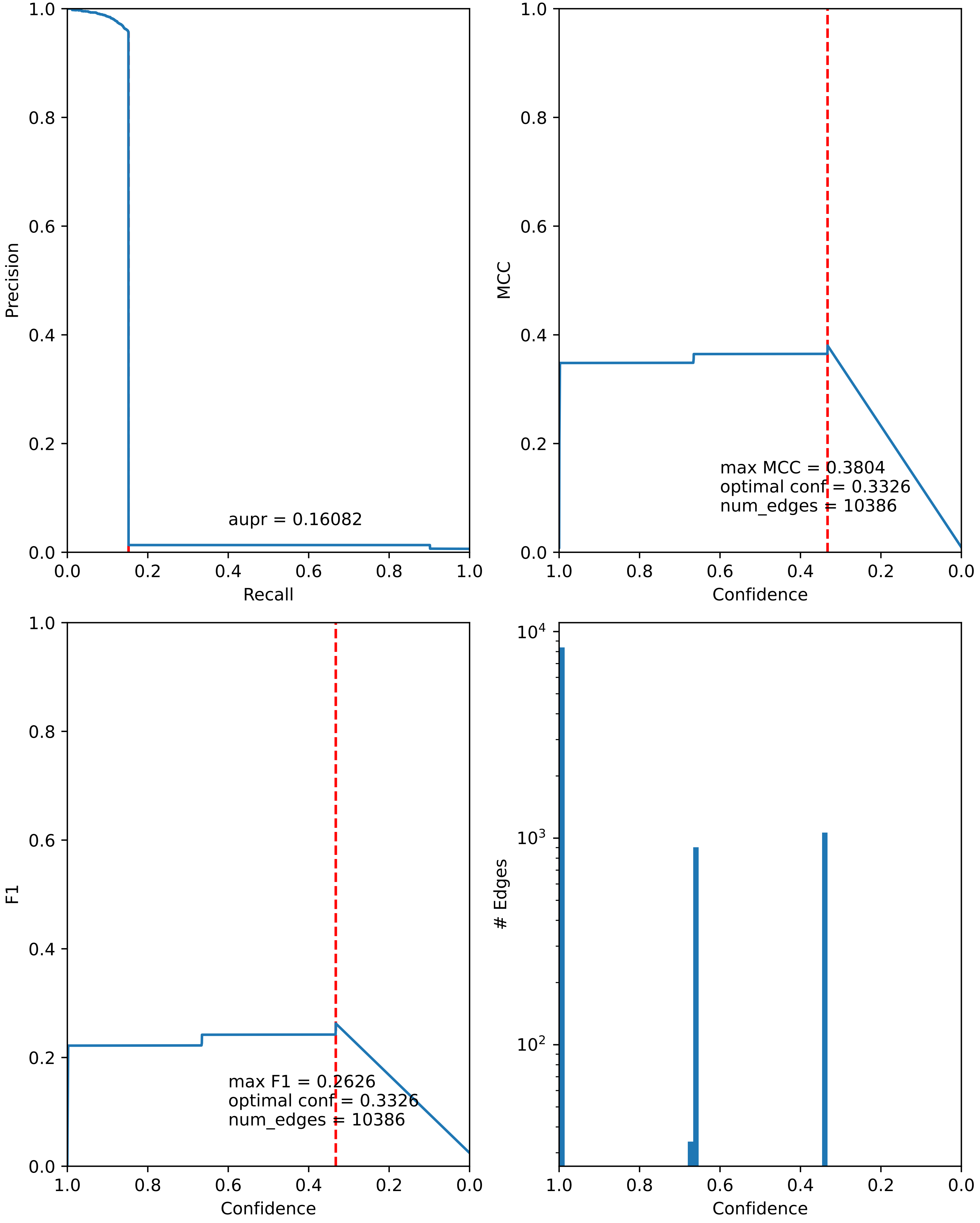

Precision
Recall
aupr = 0.16082
MCC
Confidence
max MCC = 0.3804
optimal conf = 0.3326
num_edges = 10386
F1
Confidence
max F1 = 0.2626
optimal conf = 0.3326
num_edges = 10386
# Edges
Confidence
