# Supplementary material for: High-performance single-cell gene regulatory network inference at scale: the Inferelator 3.0
Source: Bioinformatics. 2022 Feb 21;38(9):2519–28. doi: 10.1093/bioinformatics/btac117 (PMC9048651; doi:10.1093/bioinformatics/btac117)
Supplement: btac117_Supplementary_Data [file btac117_supplementary_data.zip › Bioinformatics_GJS_Supplemental_Data_2.tar.gz!/GL_MCG_1/combined_metrics.pdf]

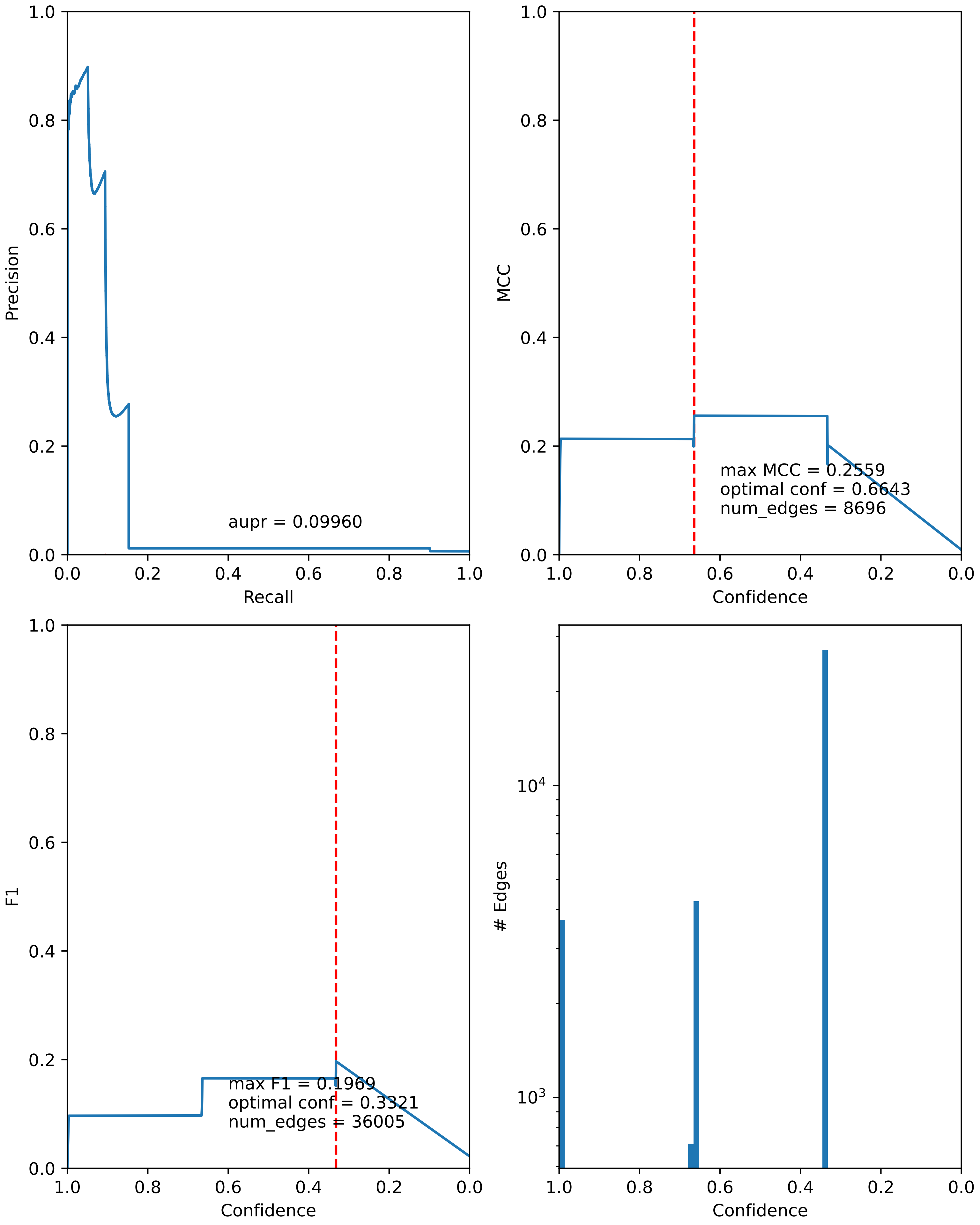

aupr = 0.09960
Precision
Recall
max MCC = 0.2559
optimal conf = 0.6643
num_edges = 8696
MCC
Confidence
max F1 = 0.1969
optimal conf = 0.3321
num_edges = 36005
F1
Confidence
Edges
Confidence
